# Solid Rocket Propellant Photo-Polymerization with an In-House LED-UV Prototype

**DOI:** 10.3390/polym15071633

**Published:** 2023-03-24

**Authors:** Andrea Galavotti, Camilla Noè, Giovanni Polizzi, Paola Antonaci, Filippo Maggi, Filippo Masseni, Dario Pastrone

**Affiliations:** 1 Department of Aerospace Science and Technology, Politecnico di Milano, Via La Masa 34, 20156 Milano, Italy; andrea.galavotti@mail.polimi.it; 2 Department of Applied Science and Technology, Politecnico di Torino, Corso Duca degli Abruzzi 24, 10129 Torino, Italy; camilla.noe@polytechnique.edu; 3 Department of Mechanical and Aerospace Engineering, Politecnico di Torino, Corso Duca degli Abruzzi 24, 10129 Torino, Italy; giovanni.polizzi@polito.it (G.P.); dario.pastrone@polito.it (D.P.); 4 Department of Structural, Geotechnical and Building Engineering, Politecnico di Torino, Corso Duca degli Abruzzi 24, 10129 Torino, Italy; paola.antonaci@polito.it

**Keywords:** composite solid propellant, polymer binder, HTPB, PBDDA, photo-polymerization, additive manufacturing

## Abstract

Composite solid propellants have used cast molding production technology for many decades, with intrinsic limitations on production flexibility, promptness, and grain geometry, as well as environmental implications on toxicity and global carbon footprint. This traditional method involves the use of toxic chemicals, has a long processing time, requires high temperature, and the products have limited geometries. To overcome those issues, different photo-curable resins have been evaluated as possible matrices. In fact, the UV-curing process is fast and has low energy consumption. The photocuring reaction parameters of six different pristine formulations were evaluated by Fourier transform infrared spectroscopy analysis. After finding the optimal curing parameters, different composites were prepared by adding 75 or 80 wt% ammonium sulfate particles used as an inert replacement for the oxidant. The thermomechanical properties and thermal resistance of the UV-cured composites were characterized via dynamic thermal-mechanical and thermogravimetric analysis. Subsequently, the mechanical properties of the inert propellants were investigated by tensile tests. The most promising resin systems for the production of solid rocket propellants were then 3D printed by an in-house developed illumination system and the obtained object micro-structure was evaluated by X-ray computed tomography.

## 1. Introduction

**Figure 1:**
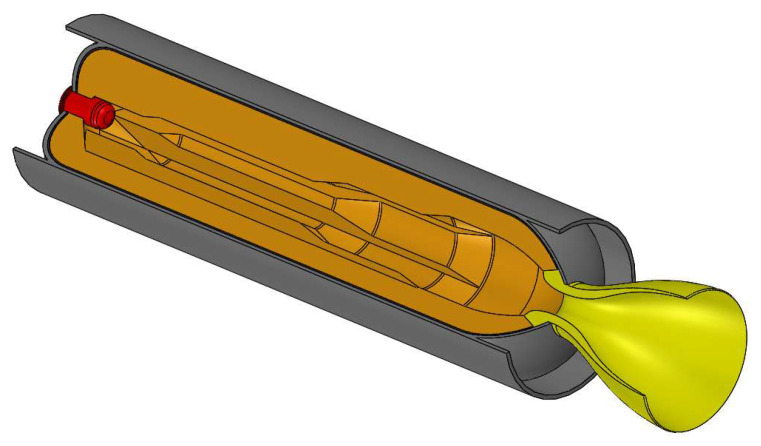
Scheme of a typical solid rocket motor: propellant grain (orange), igniter (red), insulation (black), motor case body (gray), and nozzle (yellow).

The fast production of gas with compact devices is a critical task in many applications, from thrust generation in rocket motors to airbags and fire extinguishers. One possible solution is to burn a grain or a cartridge made of solid propellant—an energetic material in which both oxidizer and fuel are mixed in solid state. Solid rocket motors (SRM) are commonly used in aerospace applications to provide thrust due to their high energy density, compactness, operational readiness, reliability, low cost, and storability [1]. An SRM (see Figure 1) basically consists of a case that typically stores the propellant and serves as a pressure vessel, an igniter, and a nozzle. Further components may be added, such as a thrust termination device, destruction system, and thrust vectoring system. The thrust history of SRMs depends on the propellant grain composition (chemical ingredients and their percentage) and manufacturing process, grain geometry and burning surfaces, nozzle type, and dimensions. Commanded extinction, throttleability, and reignition of large SRMs for space applications is actually unfeasible because of the lack of any controllable propellant flow, while it still represents a research and technological challenge for much smaller applications [2,3,4].

Composite propellants are heterogeneous energetic materials where a polymeric matrix bonds a blend of solid powders and acts at the same time as structural element and as fuel. The oxidizer is usually ammonium perchlorate (AP) or other crystalline compounds, in the amount of 60% to 90% by mass. Aluminum or other metals can be added to the propellant mixture as high energy density fuel (from 0% to 20% by mass of the total). The combustion process can be finely tuned by the addition of a small percent of ballistic modifiers (e.g., burning catalysts, retarding agents, or coolants) and/or additives, which can improve the mixing (plasticizers, wetting agents, and curing catalysts) [5].

Today, hydroxyl-terminated polybutadiene (HTPB) is one of the most widely used binders [6,7]. It is a viscous liquid prepolymer, which typical molecular weight is roughly 3000 g/mol in this kind of application. HTPB is cured through a polyaddition reaction using multifunctional isocyanates and forms a rubber-like material. Unfortunately, isocyanates are toxic compounds; some of them are classified as potential human carcinogens and are known to cause cancer in animals. Furthermore, exposure to these reagents causes lung problems, asthma, and irritation of the throat, nose, eyes, and skin [8,9].

Nowadays, in the majority of the production facilities the final shape of the grain is obtained by means of a batch-based standard mix-cast method and a forming mandrel. Continuous or semi-continuous techniques are currently under development for process optimization, production increment, and consequent cost reduction [10,11]. Sutton and Biblarz reports in [1] the basic steps of a typical batch production involving an inert binder. First, all the ingredients, with the exception of the oxidizer and the curing agent, are weighted and premixed. The obtained premixed slurry is stable and storable, although decantation of solid ingredients can occur, limiting the storage time. Before the actual grain production, final mixing with the oxidizer and curing agent takes place. The oxidizer is usually a blend of powder with different particle sizes; bimodal or trimodal distributions are often employed to obtain a better packing, better ballistic properties, and higher propellant density. At this step, the mixture is prone to fire and explosion risks, because of the presence of oxidizer, binder, and optional fuel materials (e.g., metal powders) in the slurries. The addition of the curing agent, typically a multi-functional isocyanate or a mixture, starts the polymerization process. The pot life is the time limit for the processability and casting of the slurry mixture. It is strongly dependent on the composition and the thermal treatment, and it runs between the addition of the curing agent and the moment when the viscosity becomes too high for casting. In engineering terms, the pot life is reached when specific mechanical properties of the slurry match some threshold values, such as doubling the initial viscosity or reaching the gel point. Once the slurry mixture is poured into the motor case, a mandrel is usually required to obtain the final grain geometry. Its shape is limited by demolding angles, adhesion to the final product, or the impossibility of undercut geometry (if not using a mechanized or collapsible shape). The assembly is then cured in a thermostatic oven whose dimensions introduce a further constraint on the manufacturing process. In the end, the mandrel is extracted gently and slowly, to reduce the electrostatic charge accumulation due to friction between the mandrel and the propellant surface. At this point, the shape of the grain is finalized by means of tooling, and possible imperfections (e.g., void, bubble, or non-homogeneous zone, which can lead to catastrophic failures) are identified by means of nondestructive controls, such as X-ray analysis. It is easy to understand that the risk of potential defects can increase the final cost of the propellant grain and make the development of new geometries a challenging task.

Lately, photo-polymerization processes have been proposed as a suitable alternative to standard production techniques in many different fields such as: coatings [12,13], composites [14], microelectronics [15], medicine [16], additive manufacturing [17,18,19] and more recently also in the energetic material production [20,21,22]. In particular, in the energetic material production, UV-curing technique seems to find its best fallout on optimization of charge grain production, overcoming shaping issues, and avoiding extra tooling costs, as enabler of additive manufacturing in this specific field. At the same time, a set of ancillary advantages are also present. In the photo-polymerization, the UV-light initiates the curing reaction, therefore the use of toxic thermal reagents such as isocyanates can be avoided. The pot life of grain formulations is extended since, in principle, polymerization should not start when the UV light is off. Moreover, it has a higher curing speed and a lower energy consumption with respect to thermocuring, it does not require heat addition, and it does not produce volatile compound emission (VOC). As a drawback, its applicability is limited by the depth of the cure of the formulation, which is the maximum thickness of the samples through which light can penetrate. The methodology evolved progressively over time. Xiangyang et al. patented a 3D-print-like approach for the deposition of multiple compounds, which was then followed by a two-step forming process that employed a mixture of two different prepolymers [23]. Initially, the grain shape was obtained by photocuring one of the prepolymers present in the mixture, and then the other one was thermally cured to achieve the final mechanical properties. In addition, McClain et al. proved the viability of an additive manufacturing method for the solid propellants production and successfully printed grains with a solid content of 85% [20]. In that work, two formulations containing HTPB or Illumanbond 60-7105 as a binder were compared and both thermo and UV-curing were considered. Interestingly, the authors were able to obtain solid propellants with only 30 min of processing time when the photo-polymerization technique was applied. Pastrone and co-authors patented a UV-curing methodology based on UV-A sensitive photo-initiators and presented some preliminary results [21,22]. This work demonstrated the ability to produce solid propellants with proper mechanical and physical properties. HTPB and polybutadiene diacrylate (PBDDA) were considered as binders, a UV-A (390 nm) sensitive photoinitiator was employed, and the effect of the addition of aluminum powder were investigated.

In the present paper, different photo-curable resins were carefully investigated to exploit their applicability as solid propellant binders. The curing kinetics of the different formulations was investigated by varying the amount of photo-initiator, the irradiation time, and the atmosphere. The thermal and mechanical properties of the obtained inert propellants were evaluated and compared in order to find the composites with the best characteristics for the grain production. Finally, a prototype of an illumination system was developed to enable the layer by layer fabrication of the solid propellant. The effectiveness of the prototype was tested using X-ray computed tomography on the obtained 3D solid-propellant-shaped objects printed with it.

## 2. Experimental Part

Acrylates represent typical photo-curable resins, commonly used in the form of multifunctional oligomers. They find application in thin films and in situations where rapid on-demand hardening is required, such as in the dental industry. Acrylic resins were used alone or mixed with epoxy resins in the early phases of additive manufacturing with ultraviolet (UV) cure and are still used in stereolithography and digital light processing (DLP) [24]. Chain growth with light curing occurs via free-radical polymerization, in which carbon–carbon unsaturated double bonds tend to become saturated, producing a three-dimensional cross-linked chemical structure among the functionalized oligomers. In general, this type of polymerization involves initiation, propagation, transfer, and termination steps. Photosensitive resins require an initiator providing the beginning of the radical reaction, once irradiated by UV photons [25]. Radical curing can also be obtained via a thermal process in a similar way, although a different chemical species starts the mechanism, typically a peroxide. In UV curing, activation can be triggered either by photochemical reaction, where a single compound undergoes homolitic bond cleavage, or by a photoredox process, where donor and acceptor molecules operate an electron transfer. In both cases, these reactions constitute the principle of controlled radical polymerization (CRP), where curing reactions may be tuned through light, enabling temporal and spatial control of polymer chain reactivity. Regarding thermal processing, light curing is more controllable and energy saving thanks to the instantaneous nature of radical generation [26]. Rather, the production of high-quality bulk volumes may become problematic due to the light penetration. In this respect, additive layer manufacturing is a convenient way of application thanks to layer-by-layer deposition.

### 2.1. Study Logic

**Figure 2:**
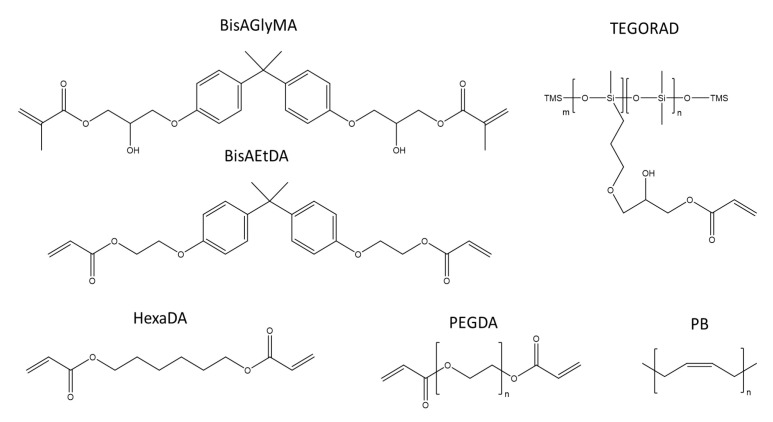
Scheme of the curing agents used in the preliminary part of this investigation.

The work was developed in two steps. Initially, the study focused on the quality evaluation of a photo-cured binder. The following photocurable resins were considered: BisAGlyMA, BisAEtDA, HexaDA, PEGDA, TEGORAD. In addition, a non-photocurable (i.e., PB) was also introduced to evaluate the possibility to produce a binder with the addition of Pentaerythritol tetrakis(3-mercaptopropionate). Their chemical structures are reported in Figure 2. The analysis mainly focused on the thermomechanical properties of the polymer matrix, as a function of the production parameters. A simulated non-aluminized propellant was prepared. The preparation was prepared using ammonium sulfate (AS) as oxidizer simulate, instead of ammonium perchlorate. These materials share similar absorption spectra in the UV range. The simulated propellants did not contain additives (e.g., iron oxide) or metal fuels (e.g., aluminum). The effect of layer superimposition was verified analyzing both monolayer and multilayer hand-deposited samples and by performing a casting of multiple batches inside a cylindrical geometry.

### 2.2. Materials

Bisphenol A glycerolate (1 glycerol/phenol) dimethacrylate (BisAGlyDMA), Bisphenol A ethoxylate diacrylate (Mn 512, EO/phenol) (BisAEtDA), 1,6-Hexanediol diacrylate (HexaDA), Polyethylene Glycole Diacrylate (Mn = 700) (PEGDA), Polybutadiene (Mn = 5000) (PB), Bis-(2,4,6-trimethylbenzoyl) phenylphosphine oxide (BAPO), and Pentaerythritol tetrakis(3-mercaptopropionate) (PTTM) were purchased from Sigma Aldrich. Ammonium sulfate (AS) was provided by Carlo Erba Reagenti. The acrylated PDMS resin “TEGORAD 2800” was gently provided by Evonik. Sunflower liquid lecithin and Bis(2-ethylhexyl) adipate were supplied by Now Foods USA and Acros Organics, respectively.

### 2.3. UV-Curable Solid Propellants Preparation

The photocurable formulations were prepared by mixing the resin with 75 or 80 wt% of AS powder (80% coarse + 20% fine) together with BAPO (photoinitiator). The only exception was the PB resin formulation, in which PTTM was also added to allow the photocuring reaction of thiol-ene to take place. Table 1 summarizes the formulations considered. After 5 min of manual stirring, a homogeneous mixture was achieved. The compound was then poured into a silicon mold for the mono-layer samples [21], whereas in the case of multi-layers, the mold was made by a number of stainless-steel shaped plates described in more detail below. The multilayer composites were prepared to evaluate propellant printability. After the UV exposure of the first single layer, another plate was stacked upon the previous one in the mold, allowing one to lay another layer and proceed again with exposure, until the desired thickness was reached. All the formulations were UV-cured using a Dymax lamp (intensity of 150 mW/cm^2^) for 1 min.

### 2.4. Cast-Cure UV Illumination Prototype

**Figure 3:**
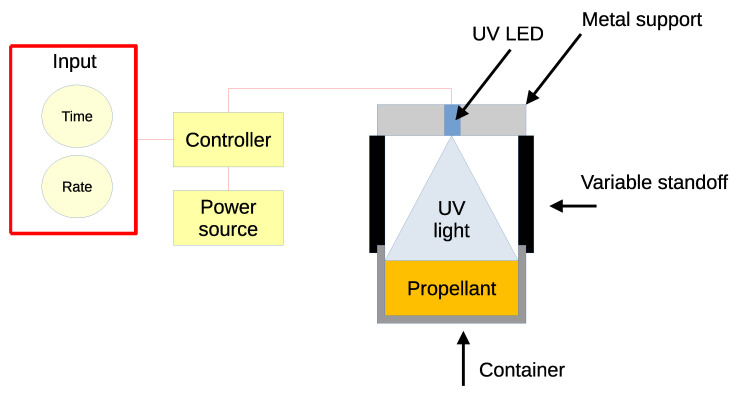
Scheme of the control unit of the UV-curing prototype.

**Figure 4:**
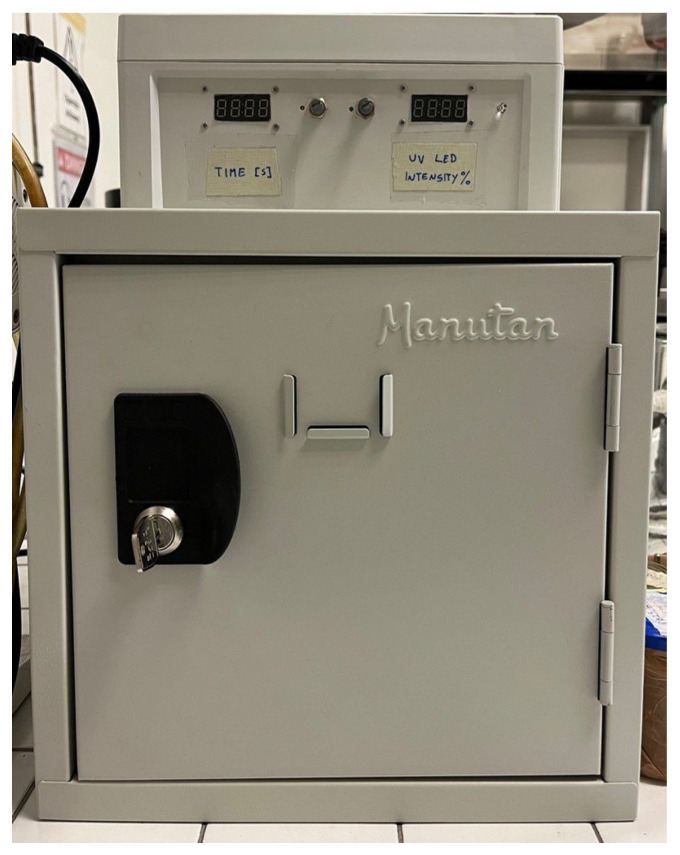
UV-curing prototype box.

A UV illumination system was developed to enable propellant casting and curing of multiple batches into a single cylindrical shape. The scheme of this prototype is reported in Figure 3. The illumination is based on one OSRAM LZ4-V4UV0R-0000, a wide angle LED emitting a nominal radiant flux of 3.3. W with a wavelength of 365 nm (measured radiant flux 3.15 W). It operates with an emission angle of 120°. The LED was mounted on an aluminum support which acted as fan-less heatsink. The system was controlled by an Arduino™UNO Rev.3 board that commanded the timing and power emission of the LED. The intensity of incident radiation reaching the sample surface was regulated on the basis of the power setting and the standoff distance. As a reference, the design point of the illumination was set to a standoff distance of 7.7 mm, 18% of power setting, granting an intensity of 12.65 W/cm^−2^. The modeling and design of the prototype considered the LED as a monochromatic Lambert source. A Delta Ohm serie HD2102 radiometer with LP471UVA probe was used to verify the setup. For safety reasons, the whole system was enclosed inside a metal box with interlock protection, depicted in Figure 4, and the operator wore Class 3 safety glasses.

This apparatus enabled the production of a sample in cylindrical configuration, following a multiple casting procedure. The sample was cast in a plastic laboratory vial having a diameter of 3 cm. Each layer was produced by pouring 5 mL of mixture from the top, after settling and illumination of the previous layer for 5 s. Each layer was about 10 mm thick, except for the first one, due to the conical shape of the vial head end. At the end of the casting, finalization of curing was performed with an illumination of 120 s on both sample edges. The sample did not need any further treatment and was ready to be tested immediately.

### 2.5. Characterization

#### 2.5.1. Fourier Transform Infrared Spectroscopy (FTIR)

All the pristine formulations were characterized by Fourier Transform Infrared Spectroscopy (FTIR), performed with a Nicolet iS 50 Spectrometer FTIR spectrometer (Perkin Elmer, Norwalk, CT, USA) in transmission mode. For each sample, 16 scans were recorded with 4 cm^−1^ resolutions. Data were processed using the software Omnic from Thermo Fisher Scientific. FTIR allowed to monitor the photo-polymerization reaction and to follow the intensity of the peaks at 1636 cm^−1^ to 1618 cm^−1^, which are attributed to the stretching vibrations of the C=C bond of the pristine resins. The double peak at 1740 cm^−1^ to 1727 cm^−1^ representing the C=O bond stretching vibration was used as an internal standard since it was not affected by the reaction. The conversion (C%) of the resins was evaluated following Equation (1)

(1) $$C_{\%}=100\;\cdot \;\left(1-\frac{\frac{A_{C=C,post}}{A_{C=C,pre}}}{\frac{A_{ref,post}}{A_{ref,pre}}}\right)$$

where $A_{C=C,pre}$ and $A_{C=C,post}$ represent the area of the C=C bond peak before and after the photo-polymerization reaction, respectively, and $A_{ref,pre}$ and $A_{ref,post}$ represent the area of the reference peak (C=O bond) before and after the photo-polymerization reaction. Different amounts of BAPO were tested for each pristine resin. The photo-cross-linking reactions were monitored at different irradiation times (10 s, 20 s, 30 s, 60 s, and 120 s) with and without an inert atmosphere (N2).

#### 2.5.2. Density Measurements

The density of the liquid resins (before the UV-irradiation) and of the cured samples (after the cross-linking) were measured by means of Archimede’s method [27]. The experiments were repeated in triplicate.

#### 2.5.3. Particle Size Distributions

A planetary ball mill was used to ground and sieve the AS raw material. Then, the size of the selected particles were measured by means of a Malvern Mastersizer 2000 through laser diffraction.

#### 2.5.4. Dynamic Thermal-Mechanical Analysis (DMTA)

Dynamic mechanical-thermal analysis (DMTA) tests were performed on the pristine and inert-loaded formulations with a Triton Technology-Tritec 2000 DMA. Samples were cooled with liquid nitrogen and measurements were run with a heating rate of 3 °C/min in tensile mode with a frequency of 1Hz. DMTA was performed to fully characterize the thermal and visco-elastic properties of the UV-cured formulations. In fact, thanks to this technique, it was possible to evaluate the storage (E’) and dissipative (E”) modulus of the material over a large temperature range. The ratio of these two moduli E”/E’ provides the parameter called Tanδ. The maximums of Tanδ correspond to the glass transition temperature $T_{g}$ of the materials.

#### 2.5.5. Thermo Gravimetric Analysis (TGA)

Thermo Gravimetric Analysis (TGA) tests were performed with a Mettler Toledo TGA/SDTA 851e to evaluate the thermal stability of the films obtained. Samples (10–12 mg) were inserted into alumina crucibles and heated at 10 °C/min from 25 °C to 900 °C with N2 flux.

#### 2.5.6. Tensile Tests

**Figure 5:**
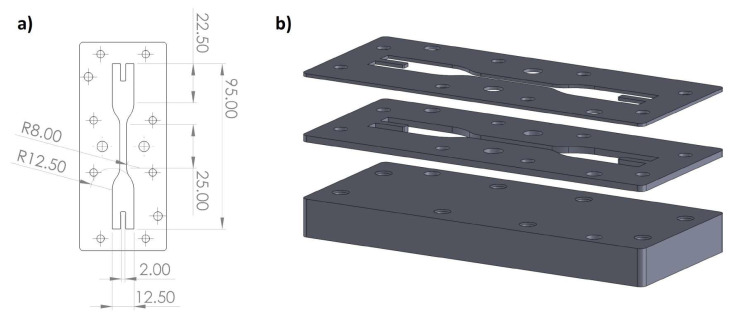
(**a**) Multilayer sample geometry (dimensions in mm); (**b**) exploded view of baseplate, 2 mm plate, and 1mm plate.

**Figure 6:**
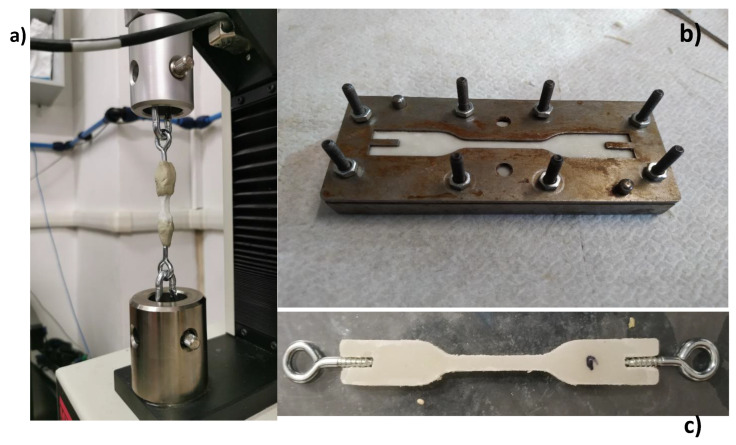
(**a**) Tensile test set-up; (**b**) baseplate and plates stacked during samples preparation; (**c**) multi-layer sample with eyelets.

The tensile tests were performed with the aid of an electro-mechanical testing machine MTS Insight 1kN Standard Length, equipped with a 1kN load cell and set to a test speed of 0.300 mm/s. Dog-bone-shaped specimens were produced to be subjected to such tensile tests and to evaluate the mechanical characteristics of the different material formulations, in terms of failure stress and Young’s modulus. The specimens were realized using either a silicon mold (in the case of mono-layer samples) or an ad hoc designed steel mold (for multi-layer samples). The latter is shown in Figure 5 and Figure 6 and allowed to stack different plates, 1 or 2 mm thick, up to 6 mm. These could be subsequently blocked with bolts and nuts on a steel base plate, allowing the layer-by-layer deposition and curing of the mix, up to reaching a final thickness of maximum 6 mm (in 6 layers of 1 mm thickness or 3 layers of 2 mm thickness). The geometry of both monolayer and multilayer samples followed the DIN_53504_1994 standard, with a slight modification adopted for the multilayer ones to facilitate clamping to the testing machine. In fact, the pneumatic grip system supplied with the machine was not suitable for clamping the samples, causing them to disintegrate at the end gripping portions. An alternative clamping system was therefore set up, by creating an articulated metal joint made of an eyelet-carabiner series. This was connected on one side directly to the mobile crosspiece of the machine and on the other to one end of the specimen, by means of a fast-hardening two-component epoxy adhesive. The same type of articulated connection was carried out to clamp the other end of the specimen to the fixed crossbar of the machine (see Figure 6). The chosen two-component epoxy glue, chemically compatible with the specimen and equipped with superior mechanical properties in terms of strength and stiffness, allowed a uniform distribution of the tensile stress along the gripped portion, while the articulated metal joints corresponding to the connections with the machine allowed to automatically achieve a perfect alignment of the specimen with the loading axis. Thus, it was possible to correctly perform the uniaxial tensile test and always locate the failure section within the central portion of the specimen. The failure stress was then calculated as a function of the max tensile force applied, while the Young’s modulus was calculated by linear interpolation of the initial linear segment of the stress strain σ−ϵ curve. Average values and their standard deviations were calculated over three samples per material formulation. The length of the thinner central portion of the dog bone specimen was used as the base length to calculate the strain ϵ.

#### 2.5.7. X-ray Computed Tomography (XCT)

A 3D non-destructive analysis was used to characterize the internal quality of the propellant cast in the plastic vial. Multilayer scanning was performed with a X-25 X-ray computed microtomography system by NSI. The system was equipped with an X-ray emitter of 160 kV potential. A full 360 ° scan was performed on the sample. The pixel pitch obtained for this scan was about 25 μm. The reconstruction was performed with the NSI proprietary software.

## 3. Results and Discussions

Different photocurable formulations were evaluated as suitable alternative binder for the production of solid propellants.

### 3.1. Optimization of the Photocuring Parameters

**Figure 7:**
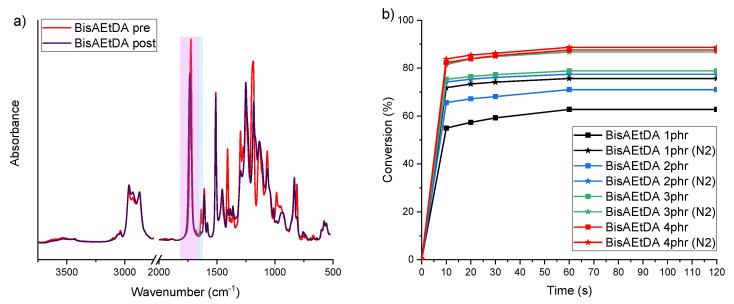
(**a**) FTIR spectra of the BisAEtDA formulation before and after UV irradiation; (**b**) FTIR C=C conversion at different irradiation time with and without inert atmosphere.

The photocuring reactivity of the pristine formulations (unloaded) was conducted using FTIR spectroscopy. In fact, FTIR analysis allows to monitor the photo-polymerization reaction by following the intensity of the peaks at 1636–1618 cm^−1^, which represent the stretching vibration of the carbon–carbon double bonds (C=C). Figure 7a presents the spectra of the BisAEtDA formulation before and after irradiation (reported here as an example) containing 4 wt% BAPO as the photoinitiator. In the graph is highlighted in blue the C=C stretching vibration and in violet the reference peak used to calculate the C%. Looking at the blue peak, it can be clearly observed that the peak after the reaction markedly decreased, which clearly indicates the success of the photo-activated cross-linking reaction. Moreover, the photocuring kinetics of the pristine resins was monitored over time with and without an inert atmosphere and with different photoinitiator (BAPO) concentrations to find the best parameters leading to highest double bond conversion (C%). For this reason, FTIR spectra were taken at different irradiation time to monitor the double bond conversion (C%). The C% over time of the BisAEtDA formulation is reported in Figure 7b as an example. The other obtained plots (C% vs. time) are reported in the Supplementary Files (Figure S1a–h) while the maximum C% are reported in Table 2. Unfortunately, the evaluation of the C=C conversion of TEGORAD could not be performed since in this case the C=C double bond peak is not clearly detectable in the spectrum. As can be observed from Figure S1a–h, all the investigated formulations showed fast reactivity and relatively small amounts of BAPO (between 2 phr and 4 phr) were always sufficient to activate the photo-cross-linking reactions. The optimized amount of BAPO of each formulation is listed in Table 2. Interestingly, no significant differences were observed between the formulation cured in an inert atmosphere or in air. This suggests that the amount of photo-initiator selected for each formulation was sufficiently high to reduce the effect of the oxygen inhibition to a negligible effect [28]. In all cases, it was possible to achieve tack-free self standing samples.

During the photo-cross-linking reaction, a density variation may be observed. In order to quantify this variation, Archimede’s method was used. The results of the comparison between the liquid resin to the corresponding cured samples are reported in Table 2. As can be observed, no significant density variations were detected.

### 3.2. Inert Formulations Characterization

The inert solid propellants were obtained by mixing the pristine resin formulations with different amounts of ammonium sulfate (AS) and subsequent irradiation with UV light. The AS salt was selected as an inert replacement for solid oxidizer (ammonium perchlorate (AP)) because it had similar solubility, polymer-bond strength, and both AS and AP crystals have been reported to be transparent in UV-visible spectra [29,30,31]. Two AS particle sizes were selected with a sieve after the grinding. Their bimodal size distributions were subsequently checked by a laser diffraction technique. The coarse and fine powder had a median particle size distribution d(0.5) of 162 μm and 13.2 μm, and a De Brouckere mean diameter D[4,3] of 200 μm and 19.1 μm, respectively. In this work an 80% coarse/20% fine AS powder was used. The photocurable formulation compositions are reported in Table 3.

The thermomechanical properties of the inert propellants were characterized by means of DMTA analysis. Unfortunately, the BisAGlyMA and HexaDA composites could not be evaluated with this technique since the obtained samples were too brittle to grip. DMTA graphs reporting Tanδ and the storage modulus as a function of temperature of the different composites are shown in Figure S2a–f. It is possible to observe the lowering of E’ in the so-called glass-transition region. The Tanδ plot of TEGORAD and PB became broader as the AS content increased indicating the formation of a more heterogeneous network, while the Tanδ curves of BisAEtDA and PEGDA remained narrow suggesting the formation of homogeneous network [32,33]. The glass transition temperatures ($T_{g}$), taken as the maximum value of the Tanδ curves, are reported in Table 3. The higher $T_{g}$ reached by the BisAGlyMA and BisAEtDA unloaded networks, with respect to the PEGDA, TEGORAD, and PB, can be attributed to the presence of aromatic rings in both the starting monomers. The HexaDA thermoset, even if the starting monomer has a linear structure, shows a high Tg due to its short molecule dimension, which leads to the formation of high cross-linked networks with a small mesh size. In turn, this leads also to an early vitrification, hence causing a lower C=C conversion as observed in Table 2. Interestingly in the composites, even if the amount of AS was really high, the Tanδ plot of BisAEtDA, PEGDA, and PB does not show high variation, implying that the mobility of the resin chain remains almost unaffected by the presence of AS. The TEGORAD composites, instead, show a marked decrease of $T_{g}$ as the AS content increased, which may be attributed to an increase in network inhomogeneity. However, for transport and storage safety, the requirement for $T_{g}$ of polymeric binders applied for standard production must be between −70 to −40 °C [21]; and as can be observed from Table 3, only few composites meet this requirement. Therefore, only a few can be considered suitable for the production of solid propellants.

Subsequently a TGA analysis was performed to evaluate the thermal stability of the obtained composites. The TGA graphs are reported in Figure S3a–f, while the data extrapolated from the curves, the initial degradation temperature ($T_{5\%}$) i.e., the temperature at which the sample loses 5% of its original mass, the maximum degradation temperature ($T_{peak}$) and the char residue % are reported in Table 4. All the TGA curves of the pristine binders, except HexaDA, show an initial small slope drop before the big slope change, representing the major decomposition step. This initial drop can be attributed to a primary depolymerization and to the degradation of the photo-initiator coupled with the degradation mechanism associated to it [34]. As can be observed in Table 4, at the end of pyrolysis the char residues of the binder samples are generally low (less than 10%) except for the BisAGlyMA (21%) and HexaDA (11%) samples. Interestingly, the maximum degradation temperatures $T_{peak}$ achieved by the binders are in the same order of magnitude as the ones previously reported in the literature for other solid propellants [35,36]. In general, samples constituted by only the binder can be considered more thermally stable than the ones containing AS since they start to degrade later (>$T_{5\%}$) and possess a higher maximum degradation temperature (>$T_{peak}$). However, a detailed investigation on the thermal stability of the loaded samples in not reported here since it is cannot be considered representative of the behavior of the final propellant.

#### 3.2.1. Mechanical Testing

Tensile tests were performed to address materials’ mechanical properties for every inert-loaded formulation. Tensile test results for the mono-layer samples, i.e., Young’s modulus $E_{t}$ and tensile strength $\sigma_{m}$, are summarized in Table 5.

Both the Young’s modulus and the tensile strength values are slightly lower than the ones reported in the literature for the HTPB cured with isocyanate. However, the results are similar to UV-cured HTPB with thiol addition, especially for PB resins, which have a higher molecular weight and better powder distribution compared to the former. Thiol plasticizing effects could be addressed for softening and these effects are still present in the latest binder formulation; the possibility of adding new components to the formulation could be evaluated to overcome this issue.

Tensile tests were also performed on the multilayer samples to evaluate the effect of layer–layer adhesion on the global mechanical properties under tension. The testing equipment, procedure, and settings were the same as those for monolayer samples. However, due to the more complex preparation and curing process, multilayer tensile tests were performed only on PB and TEGORAD loaded formulations, which showed appropriate values of $T_{g}$ in the previously performed DMTA (as reported in Table 3). Results of the tensile tests on multilayer samples are mentioned in Table 6.

Multilayer testing showed a reduction in strength compared to monolayer samples, particularly for PB resins. Possible imperfections in the adhesion between different layers could produce this effect, due to the handmade sample production procedure. Layer adhesion could benefit from controlled mechanical deposition and fine tuned time exposure. Hence, it is expected that further analysis on samples produced with controlled procedures is expected to highlight a better behavior for multilayer polymers.

#### 3.2.2. X-Ray Computed Tomography

**Figure 8:**
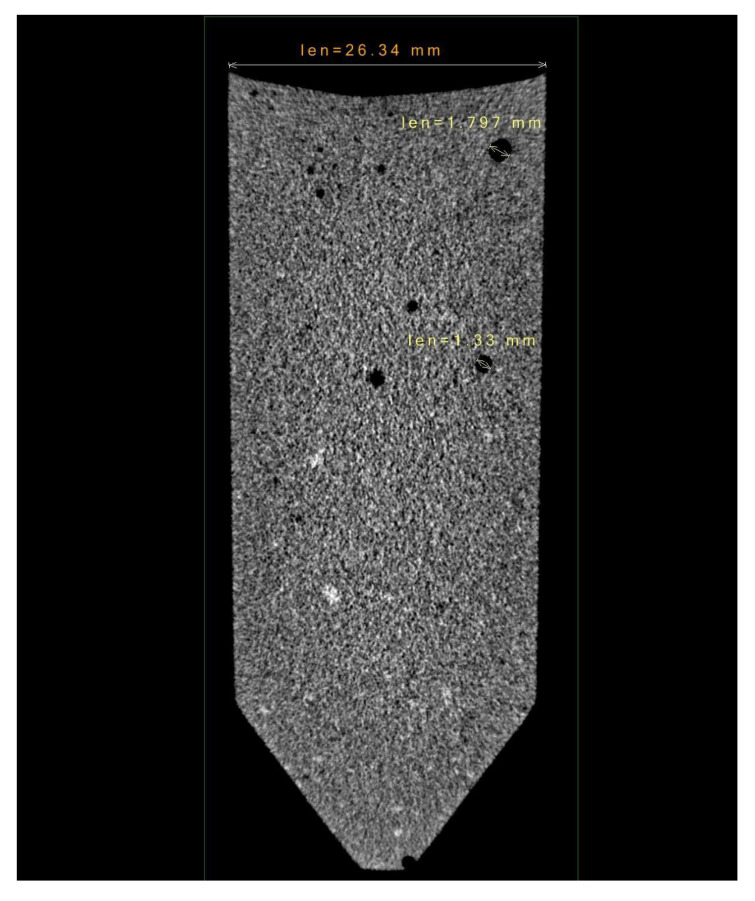
X-ray computed tomography. The scan resolution is 60 μm^3^/voxel.

**Figure 9:**
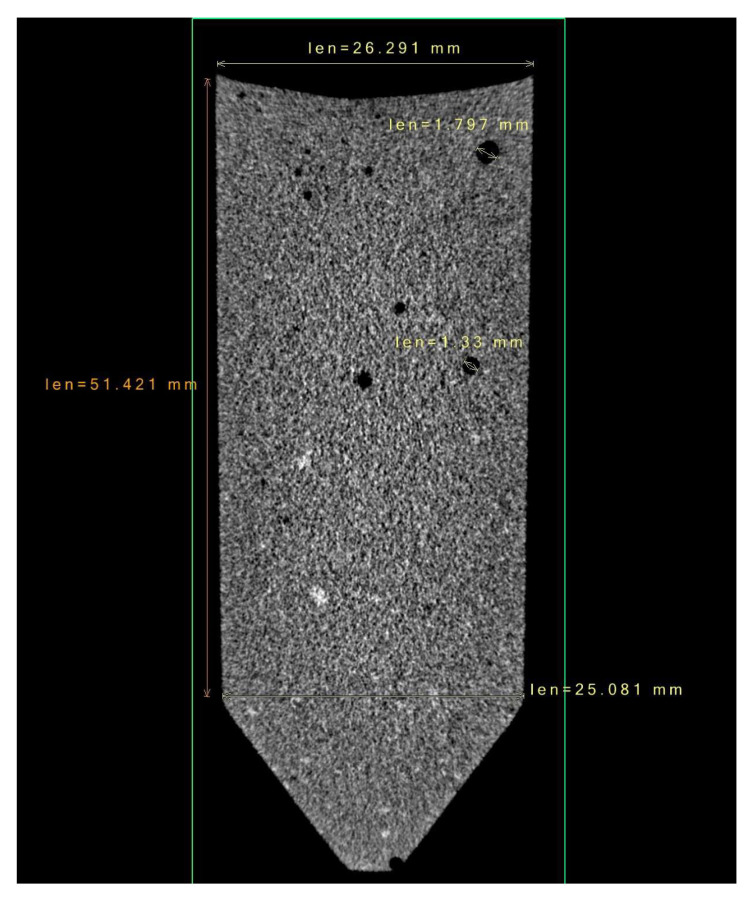
X-ray computed tomography. The scan resolution is 60 μm^3^/voxel.

The production of a cylindrical sample with multiple layers was performed using the illumination system. Figure 8 and Figure 9 demonstrate the adhesion of different layers. In each layer, 5 mL of compound was poured into the vial from the top, without specific procedures, under laboratory environmental conditions. The resulting layers were 0.85 mm thick at the nominal inner diameter of the vial, which was 26 mm. However, the vial was slightly conical (see the measurements in Figure 8); thus, the layers at the bottom resulted to be slightly thicker than the layers at the top. After each deposition, a 5 s exposure was performed. As the scan reveals, layers are not detectable in the stack, demonstrating a perfect adhesion. A small number of bubbles is also present, caused by the absence of vacuum treatment.

## 4. Conclusions

A photocuring technique was demonstrated to be a feasible method for the production of nonaluminized solid rocket propellants. Different UV-curable binders were investigated as possible matrices for the highly loaded composites. All of them showed fast reactivity with a relatively low amounts of photo-initiator, as proven by Fourier transform infrared spectroscopy. Subsequently, the resins were mixed with ammonium sulfate powder at different concentrations to produce inert solid propellants. Depending on the chemical structure of the starting material (presence of aromatic rings, chain dimensions), it was possible to obtain a wide range of thermomechanical properties, with glass transition temperature $T_{g}$ ranging from −107 to 138 °C. Moreover, all the tested composites possessed comparable heat resistance with the ones commonly used for the production of solid propellants. The mechanical properties achieved by the composites still highlight lower performance compared to those of standard isocyanate-cured compositions, although the presented materials constitute an initial approach, still to be refined and optimized, with margins of improvement. Among the resins evaluated, only TEGORAD and Polybutadiene matched the typical storage requirements for solid propellants, characterized by a very low $T_{g}$. Therefore, only the tensile properties of those two resins were further tested in multilayer configurations. Finally, to evaluate the applicability of using UV-curable resins in solid rocket propellants production, an illuminating prototype system was developed and used to prepare a PB-based simulate sample using multiple casts. The internal quality of the sample, verified by microtomography, demonstrated good adhesion between the layers and suitability of the process for this grain preparation method, even using a nonfunctional polymer, without isocyanates. The potential applicability of UV-curing additive manufacturing technique to produce solid rockets propellants with a striking reduction of toxic compounds and energy consumption is envisioned. Furthermore, the additive manufacturing technique may lead to the development of innovative complex grain geometries with enhanced propellant performances.

## Figures and Tables

**Table 1:** Composition of the formulations produced.

Formulation	Resin %	AS (wt%)	BAPO (phr)
**BisAGlyMA**	100	0	3
**25BisAGlyMA-75AS**	25	75	3
**20BisAGlyMA-80AS**	20	80	3
**BisAEtDA**	100	0	4
**25BisAEtDA-75AS**	25	75	4
**20BisAEtDA-80AS**	20	80	4
**HexaDA**	100	0	4
**25HexaDA-75AS**	25	75	4
**20HexaDA-80AS**	20	80	4
**PEGDA**	100	0	2
**25PEGDA-75AS**	25	75	2
**20PEGDA-80AS**	20	80	2
**TEGORAD**	100	0	3
**25TEGORAD-75AS**	25	75	4
**20TEGORAD-80AS**	20	80	4
**PB**	100	0	4
**25PB-75AS**	25	75	4
**20PB-80AS**	20	80	4

**Table 2:** Optimized BAPO amount for each formulation and density evaluation. The solvents used for the density investigation were: distilled water (H_2_O_*di*_) for BisAGlyMA and BisAEtDA, and ethyl alcohol (EtOH) for all others.

Formulation	BAPO (phr)	C%	$\rho_{\mathrm{liq}}$	$\rho_{\mathrm{cured}}$
			g/cm^3^	g/cm^3^
**BisAGlyMA**	3	59	1.16	1.20 ± 0.04
**BisAEtDA**	4	89	1.12	1.17 ± 0.03
**HexaDA**	4	54	1.00	1.14 ± 0.01
**PEGDA**	2	83	1.13	1.15 ± 0.08
**TEGORAD**	2	/	0.98	0.94 ± 0.02
**PB**	4	83	0.93	0.94 ± 0.04

**Table 3:** Formulations composition and glass transition temperature ($T_{g}$) of the obtained cross-linked samples.

Formulation	Resin %	AS (wt%)	$T_{g}$ (°C)
**BisAGlyMA**	100	0	138
**25BisAGlyMA-75AS**	25	75	/
**20BisAGlyMA-80AS**	20	80	/
**BisAEtDA**	100	0	69
**25BisAEtDA-75AS**	25	75	66
**20BisAEtDA-80AS**	20	80	62
**HexaDA**	100	0	92
**25HexaDA-75AS**	25	75	/
**20HexaDA-80AS**	20	80	/
**PEGDA**	100	0	−32
**25PEGDA-75AS**	25	75	−30
**20PEGDA-80AS**	20	80	−31
**TEGORAD**	100	0	−107
**25TEGORAD-75AS**	25	75	−38
**20TEGORAD-80AS**	20	80	−41
**PB**	100	0	−68
**25PB-75AS**	25	75	−70
**20PB-80AS**	20	80	−56

**Table 4:** Thermal properties of the composites (inert).

Formulation	$T_{5\%}$	$T_{\mathrm{peak}}$	Char Residue
-	°C	°C	%
**BisAGlyMA**	375	421	21
**25BisAGlyMA-75AS**	294	335	14
**20BisAGlyMA-80AS**	307	332	15
**BisAEtDA**	316	436	8
**25BisAEtDA-75AS**	297	340	19
**20BisAEtDA-80AS**	281	324	13
**HexaDA**	392	428	11
**25HexaDA-75AS**	290	390	11
**20HexaDA-80AS**	294	391	9
**PEGDA**	366	411	6
**25PEGDA-75AS**	269	316	15
**20PEGDA-80AS**	278	320	10
**TEGORAD**	376	490	0.8
**25TEGORAD-75AS**	290	406	6
**20TEGORAD-80AS**	296	410	6
**PB**	362	461	3
**25PB-75AS**	291	435	9
**20PB-80AS**	292	434	9

**Table 5:** Monolayer tensile testing results (inert).

Formulation	$E_{t}$	$\sigma_{m}$
-	MPa	MPa
**25BisAGlyMA-75AS**	185.25 ± 4.9	3.33 ± 0.3
**20BisAGlyMA-80AS**	174.92 ± 39.1	3.2 ± 0.1
**25BisAEtDA-75AS**	347.92 ± 53.7	7.06 ± 0.2
**20BisAEtDA-80AS**	362.64 ± 23.3	6.03 ± 0.2
**25HexaDA-75AS**	292.8 ± 22.2	4.43 ± 0.3051
**20HexaDA-80AS**	256.98 ± 41.6	3.80 ± 0.3
**25PEGDA-75AS**	28.19 ± 16.4	0.77 ± 0.1
**20PEGDA-80AS**	22.56 ± 3.1	0.64 ± 0.1
**25TEGORAD-75AS**	5.28 ± 1.8	0.22 ± 0.01
**20TEGORAD-80AS**	11.42 ± 1.7	0.33 ± 0.01
**25PB-75AS**	11.9925 ± 2.97	0.6 ± 0.1
**20PB-80AS**	27.05 ± 2.5	0.64 ± 0.04

**Table 6:** Multilayer tensile testing results (inert).

Formulation	$E_{t}$	$\sigma_{m}$
-	MPa	MPa
**25TEGORAD-75AS**	13.06 ± 1.3	0.30 ± 0.03
**20TEGORAD-80AS**	19.21 ± 2.0	0.28 ± 0.02
**25PB-75AS**	18.75 ± 5.48	0.47 ± 0.02
**20PB-80AS**	16.94 ± 3.9	0.39 ± 0.1

## Data Availability

Data is contained within this article and its Supplementary Materials.

## Supplementary Materials

The following supporting information can be downloaded at: https://www.mdpi.com/article/10.3390/polym1010000/s1. Figure S1a: FTIR spectra of the BisAGlyMA formulation before and after the UV irradiation; Figure S1b: FTIR C=C conversion of the BisAGlyMA formulation at different irradiation time with and without inhert atmosphere; Figure S1c: FTIR spectra of the HexaDA formulation before and after the UV irradiation; Figure S1d: FTIR C=C conversion of the HexaDA formulation at different irradiation time with and without inert atmosphere; Figure S1e: FTIR spectra of the PEGDA formulation before and after the UV irradiation; Figure S1f: FTIR C=C conversion of the PEGDA formulation at different irradiation time with and without inhert atmosphere; Figure S1g: FTIR spectra of the PB formulation before and after the UV irradiation; Figure S1h: FTIR C=C conversion of the PB formulation at different irradiation time with and without inert atmosphere; Figure S2a: DMTA graphs of BisAGlyMA formulation; Figure S2b: DMTA graphs of BisAEtDA formulations; Figure S2c: DMTA graphs of HexaDA formulation; Figure S2d: DMTA graphs of PEGDA formulations; Figure S2e: DMTA graphs of TEGORAD formulations; Figure S2f: DMTA graphs of PB formulations; Figure S3a: TGA graphs of BisAGlyMA formulation; Figure S3b: TGA graphs of BisAEtDA formulations; Figure S3c: TGA graphs of HexaDA formulation; Figure S3d: TGA graphs of PEGDA formulations; Figure S3e: TGA graphs of TEGORAD formulations; Figure S3f: TGA graphs of PB formulations.

## Abbreviations

The following abbreviations are used in this manuscript:

AP	Ammonium Perchlorate
AS	Ammonium Sulfate
DMTA	Dynamic Thermal-Mechanical Analysis
FTIR	Fourier Transform Infrared Spectroscopy
HTPB	Hydroxyl Terminated Polybutadiene
PB	Polybutadiene
PDMS	Polydimethylsiloxane
BAPO	Bis-(2,4,6-trimethylbenzoyl) phenylphosphine oxide
SRM(s)	Solid Rocket Motor(s)
TGA	TermoGravimetric Analysis
UV	UltraViolet
**Nomenclature**	
*A*	Area
$C_{\%}$	Resins Conversion
$E_{t}$	Young’s modulus, MPa
*T*	temperature, °C
ρ	density, kg/m^3^
$\sigma_{m}$	tensile strength, MPa
**Subscripts**	
5%	Initial degradation
C=C,post	C=C bond peak after photo-polymerization reaction
C=C,pre	C=C bond peak before photo-polymerization reaction
*g*	glass transition
peak	peak, maximum degradation
ref	reference
