# Supplementary figures and images for: Solid Rocket Propellant Photo-Polymerization with an In-House LED-UV Prototype
Source: Polymers (Basel). 2023 Mar 24;15(7):1633. doi: 10.3390/polym15071633 (PMC10097351; doi:10.3390/polym15071633)

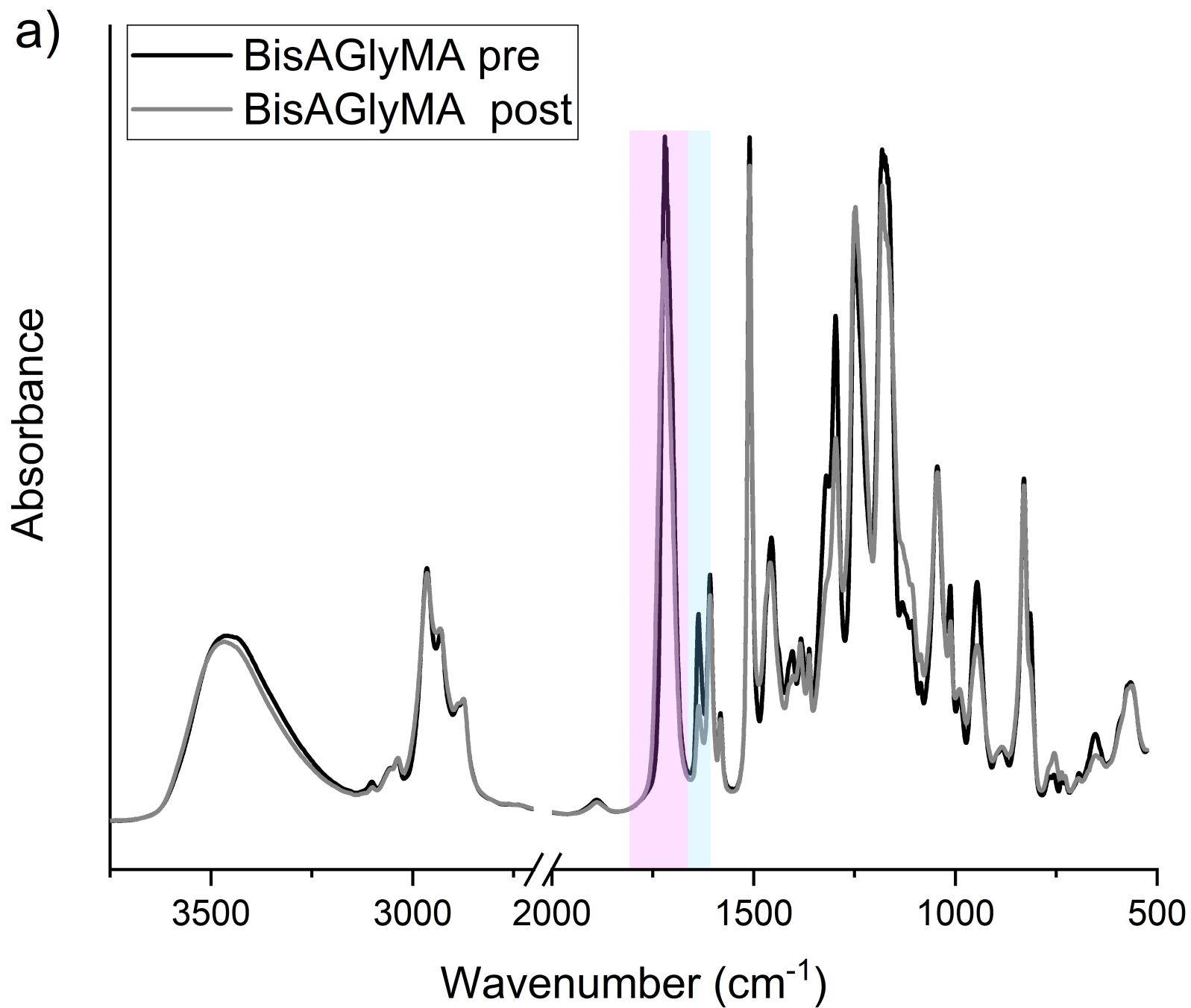

Supplement: Supplementary file 1 [file polymers-15-01633-s001.zip › Fig.S1a.pdf]

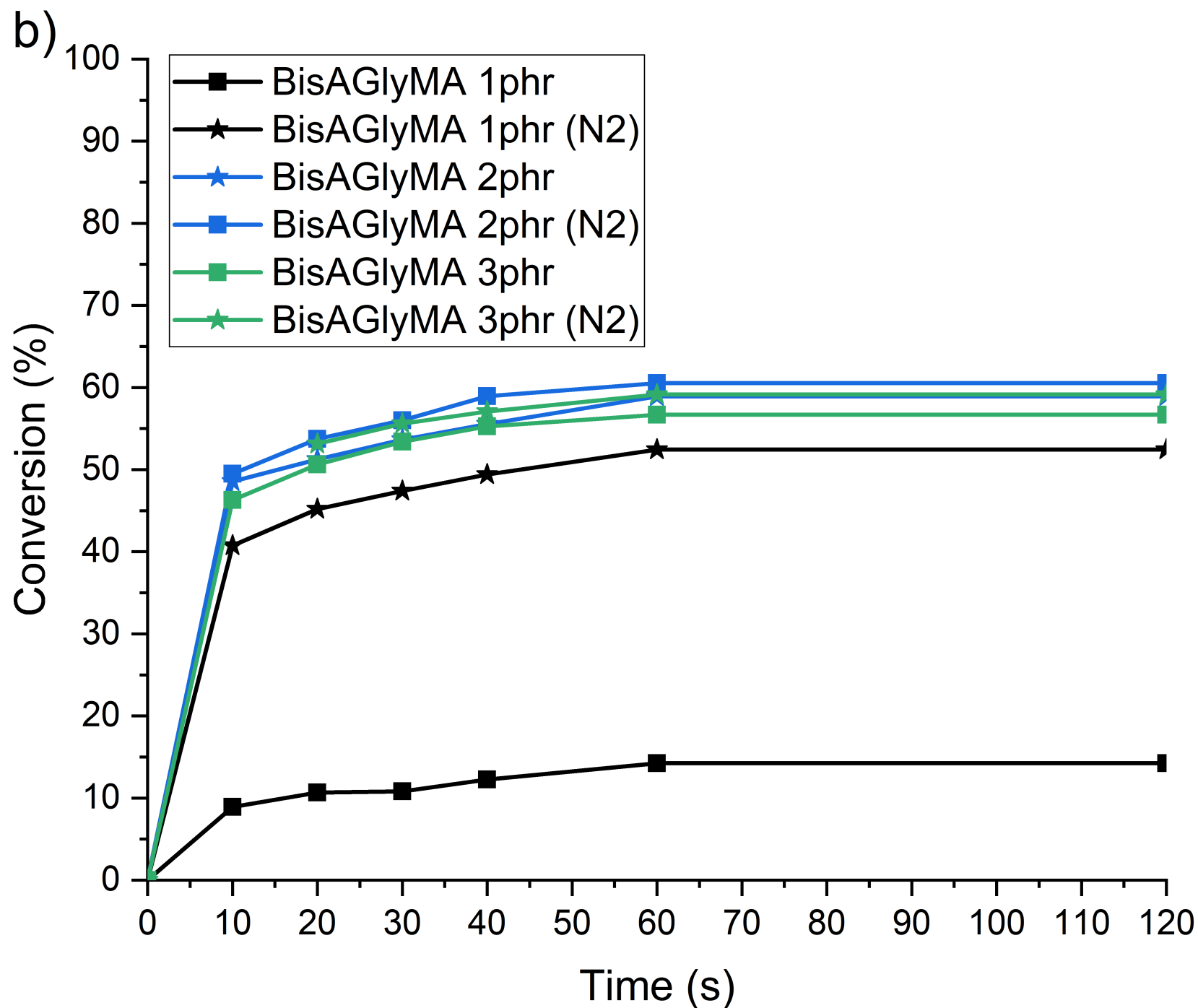

Supplement: Supplementary file 1 [file polymers-15-01633-s001.zip › Fig.S1b.pdf]

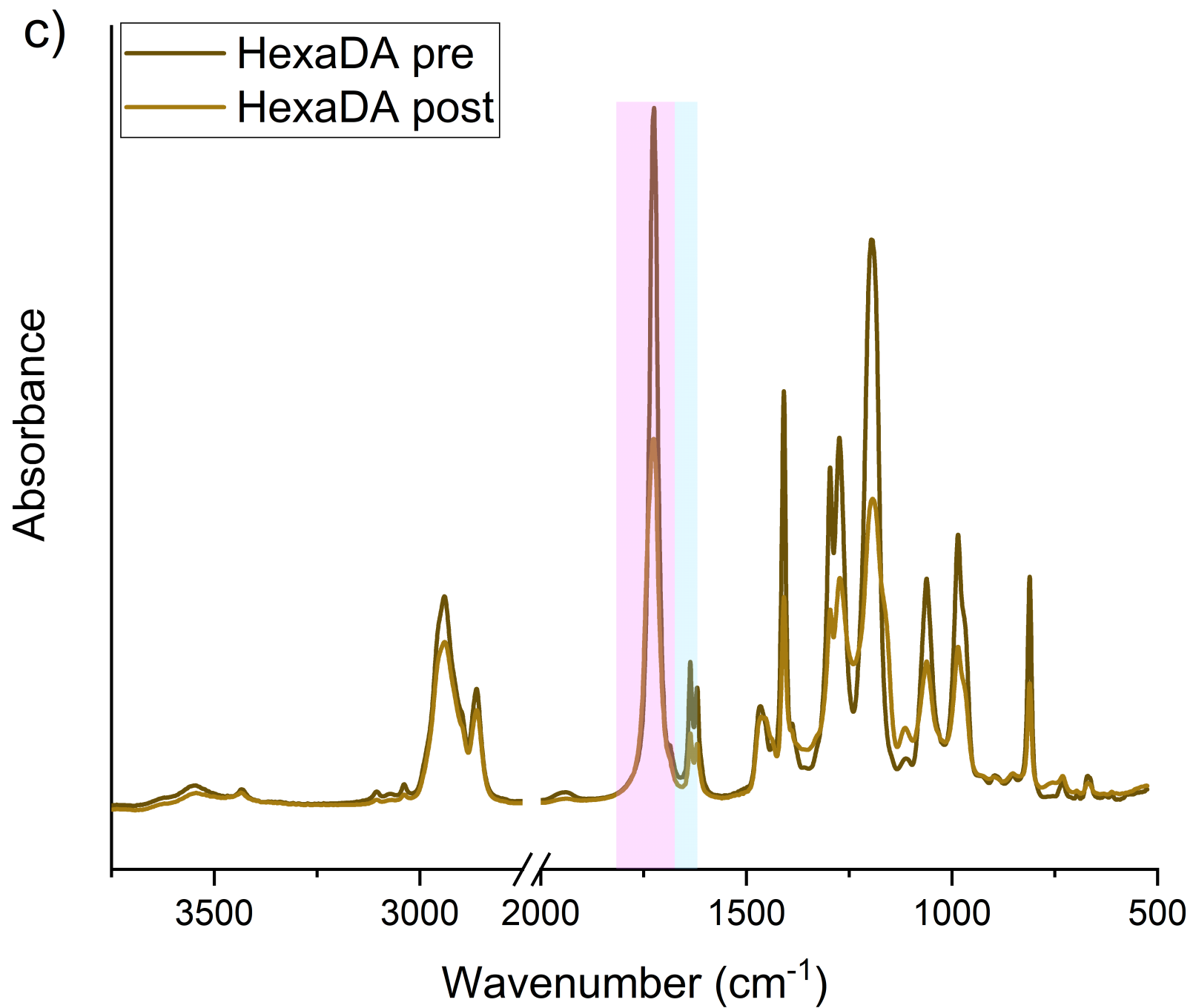

Supplement: Supplementary file 1 [file polymers-15-01633-s001.zip › Fig.S1c.pdf]

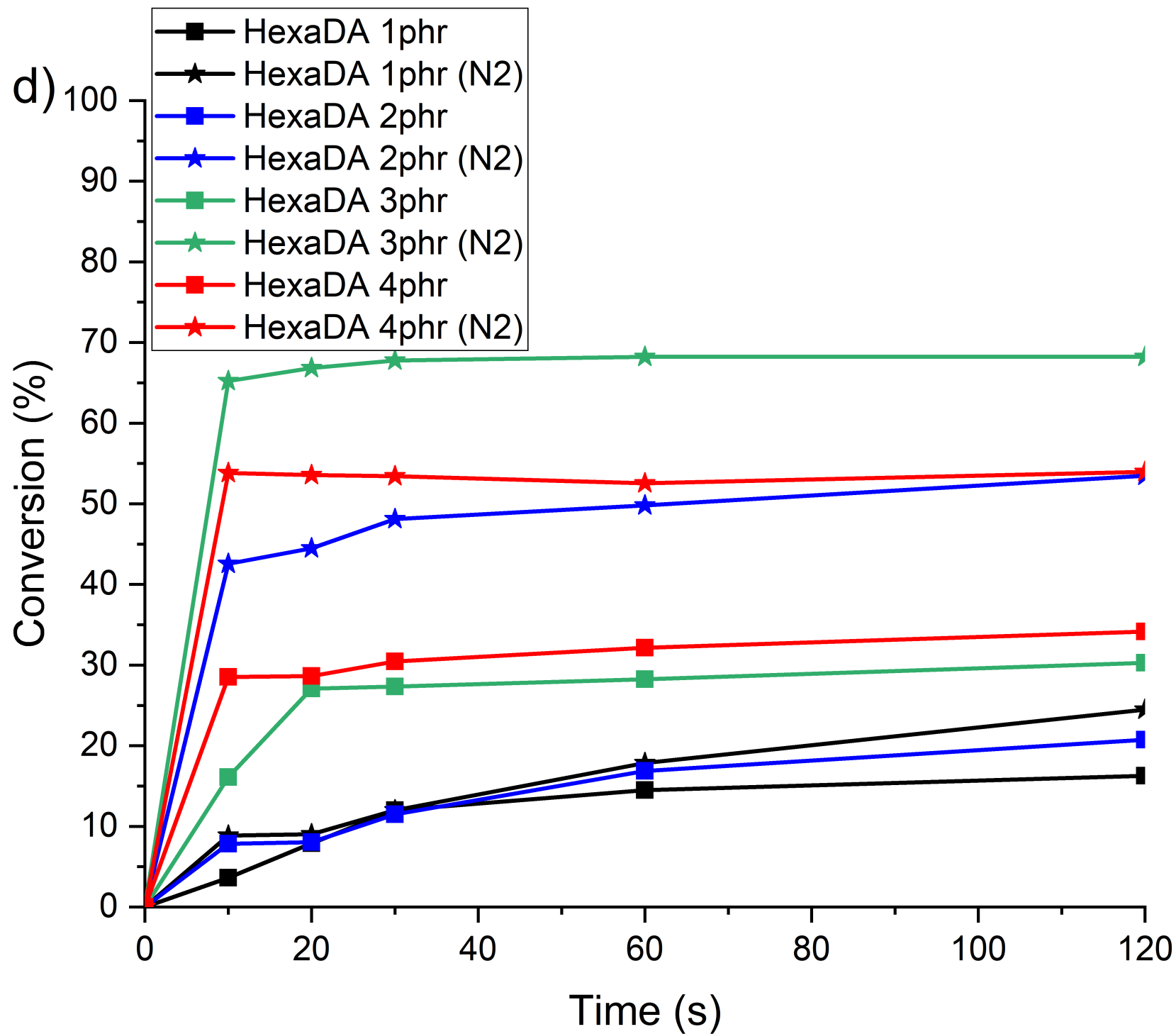

Supplement: Supplementary file 1 [file polymers-15-01633-s001.zip › Fig.S1d.pdf]

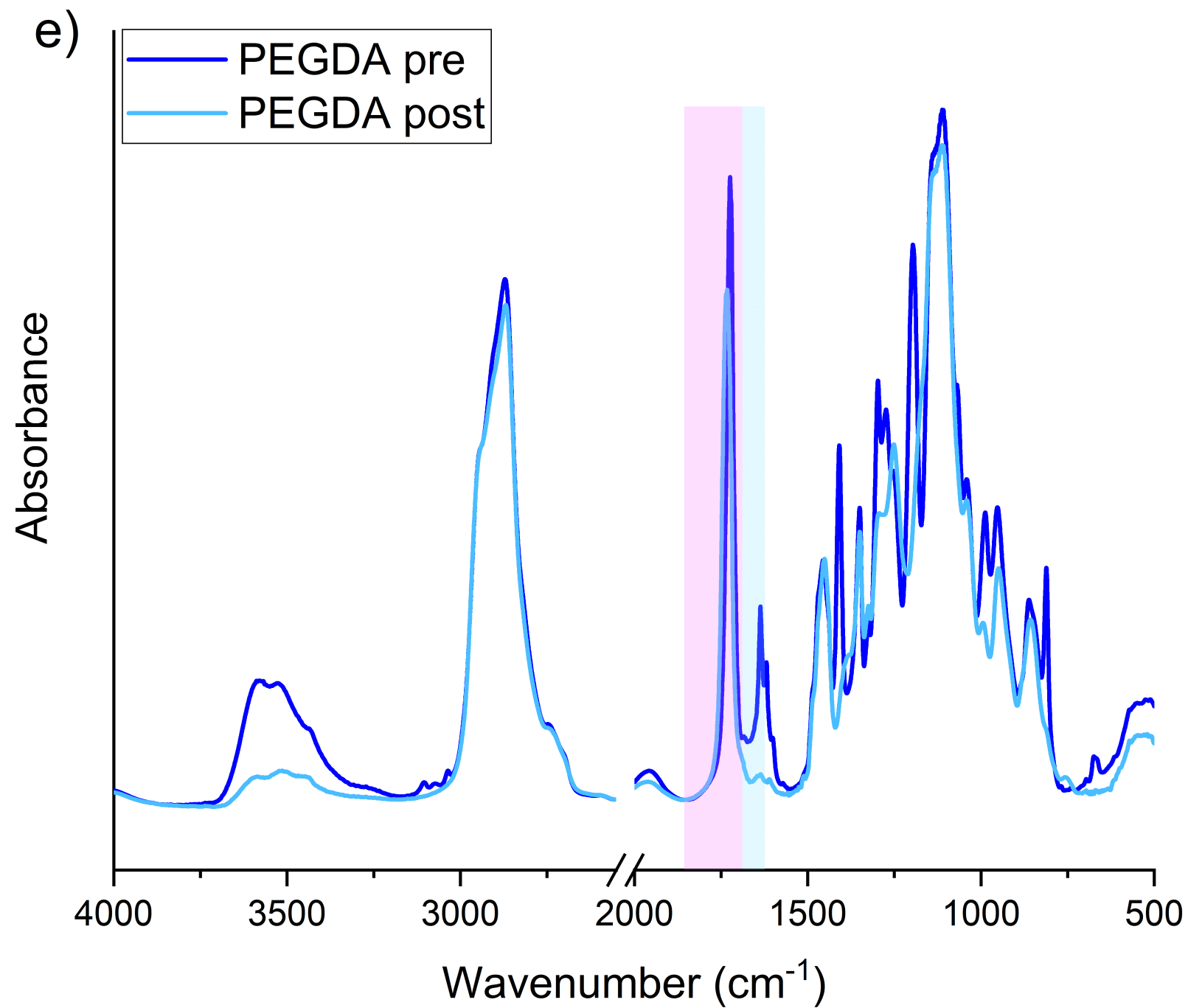

Supplement: Supplementary file 1 [file polymers-15-01633-s001.zip › Fig.S1e.pdf]

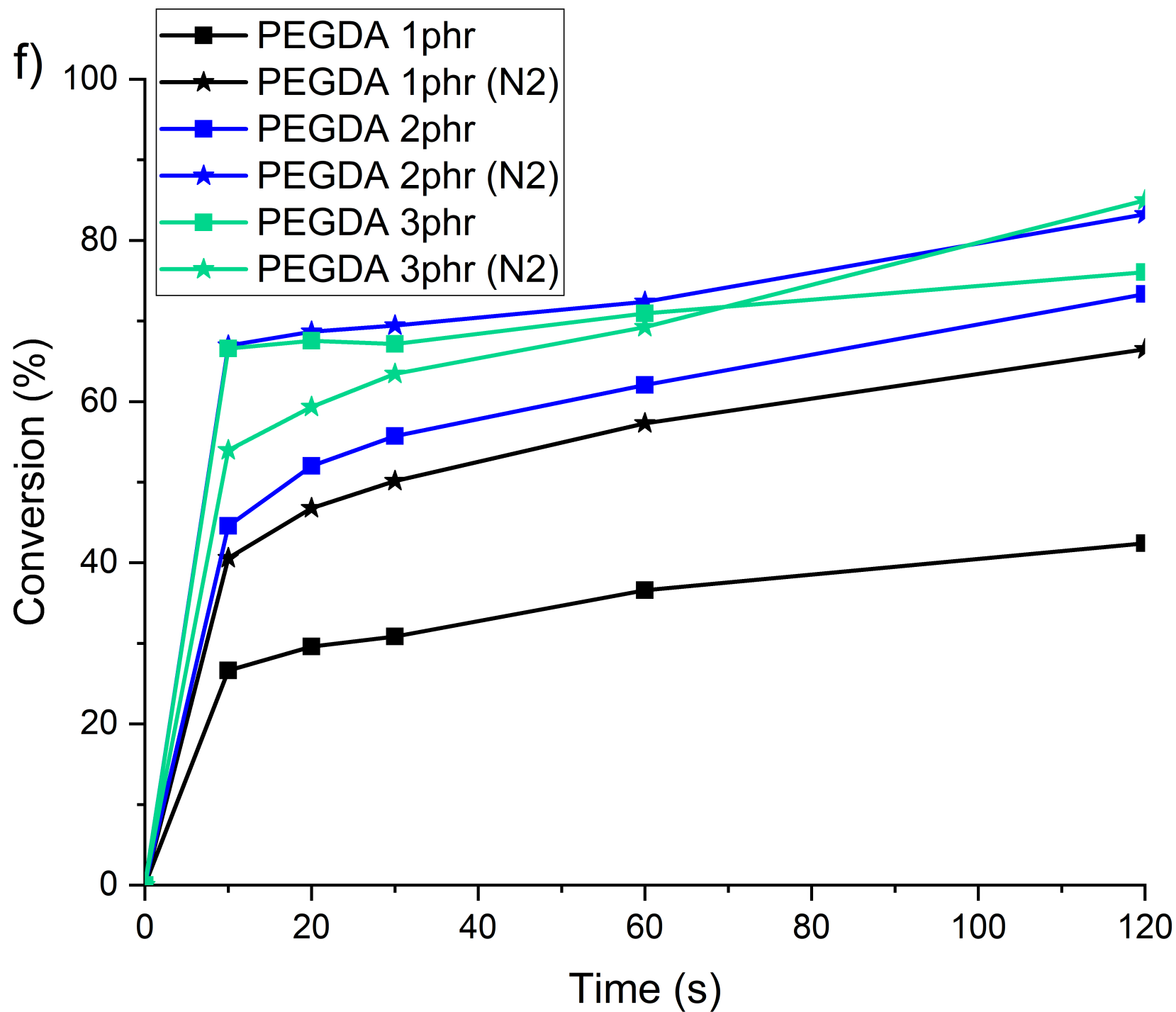

Supplement: Supplementary file 1 [file polymers-15-01633-s001.zip › Fig.S1f.pdf]

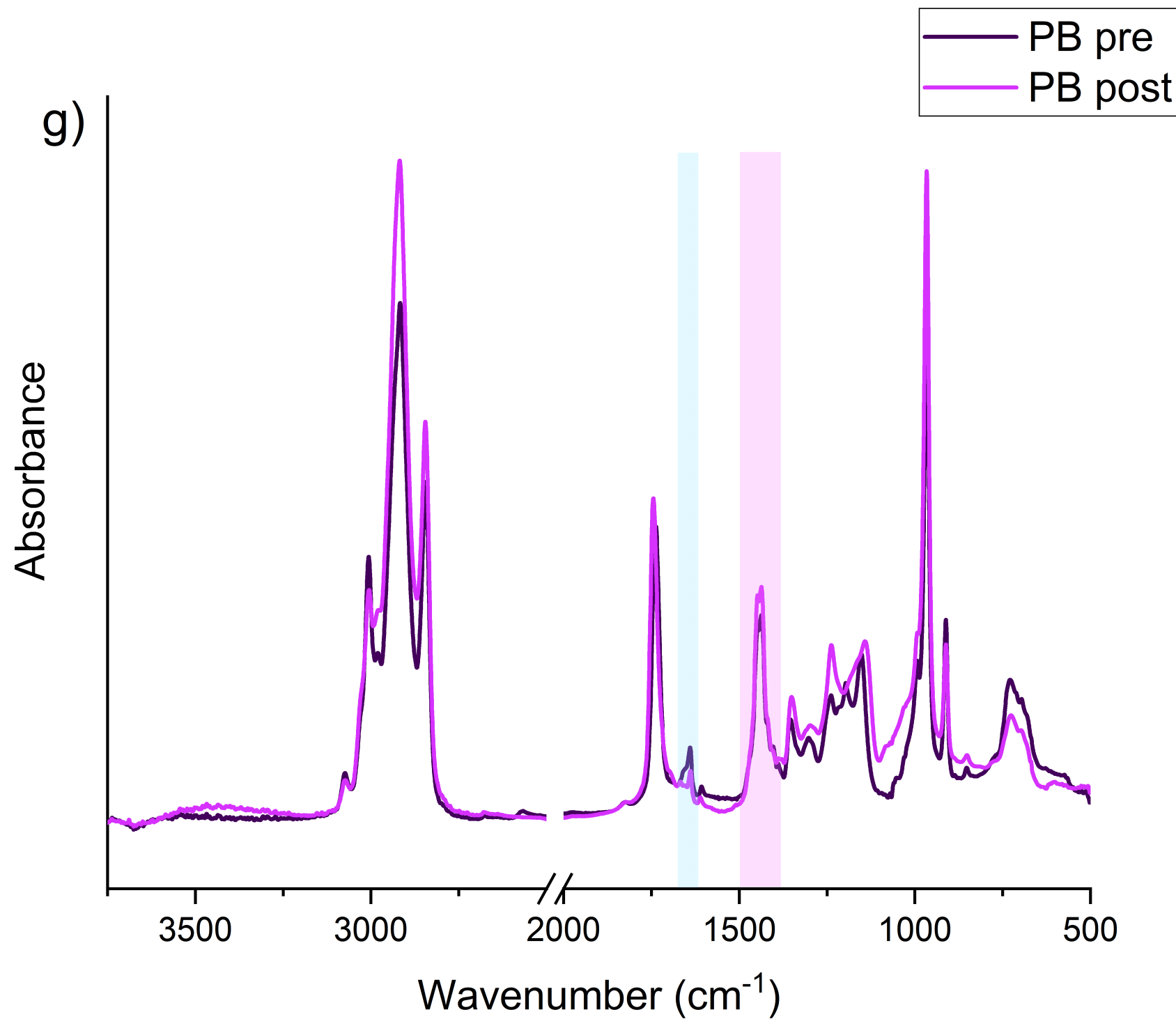

Supplement: Supplementary file 1 [file polymers-15-01633-s001.zip › Fig.S1g.pdf]

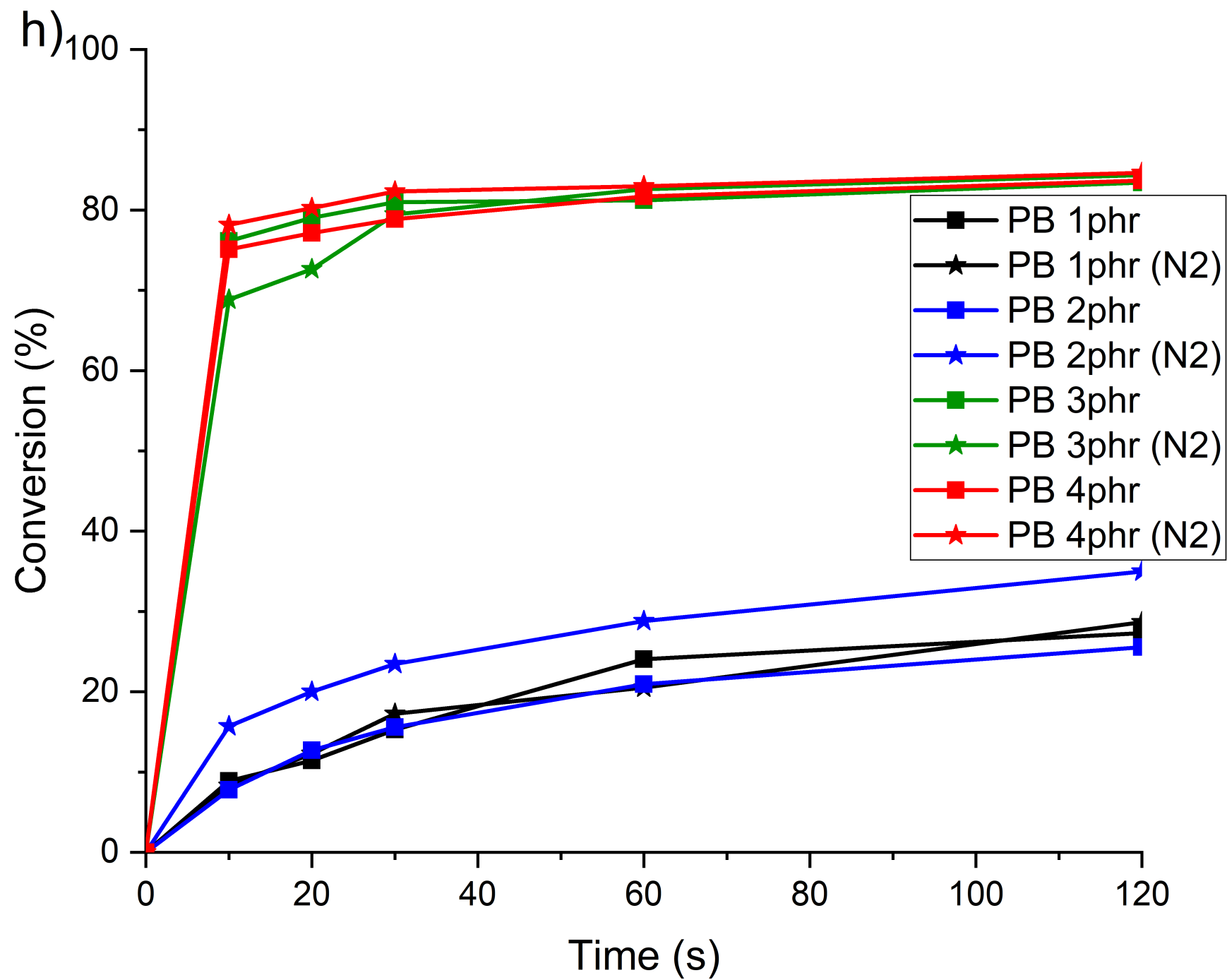

Supplement: Supplementary file 1 [file polymers-15-01633-s001.zip › Fig.S1h.pdf]

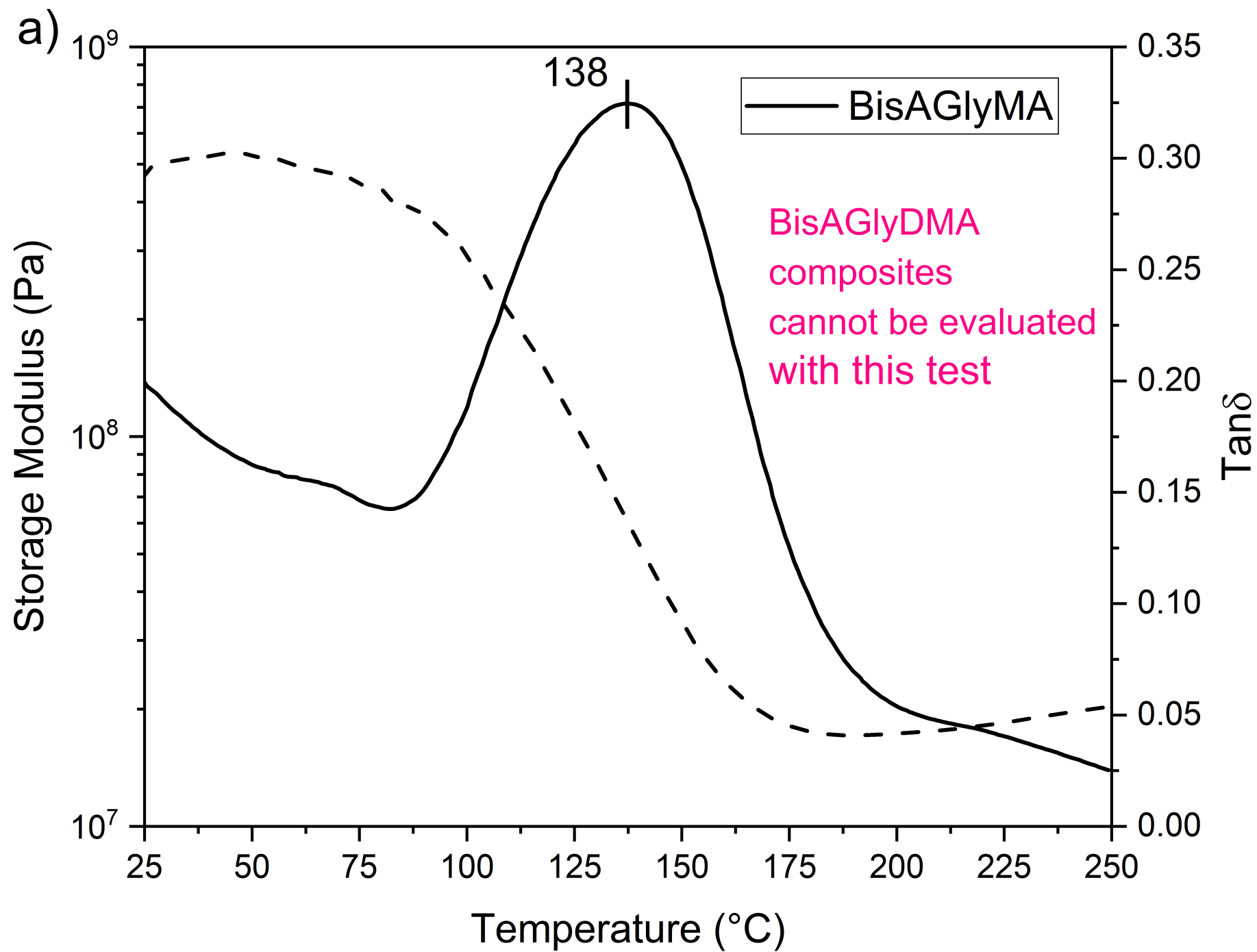

Supplement: Supplementary file 1 [file polymers-15-01633-s001.zip › Fig.S2a.pdf]

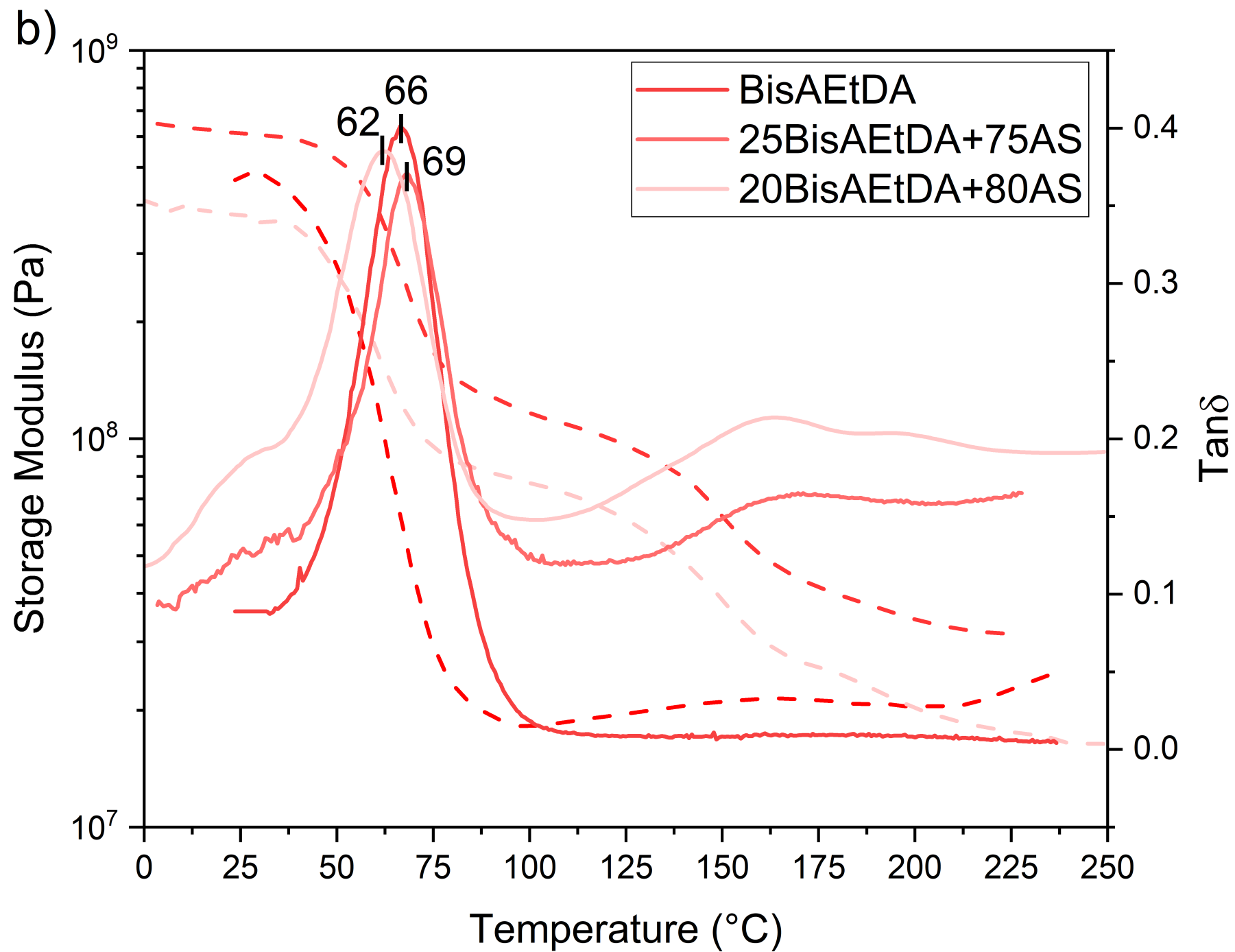

Supplement: Supplementary file 1 [file polymers-15-01633-s001.zip › Fig.S2b.pdf]

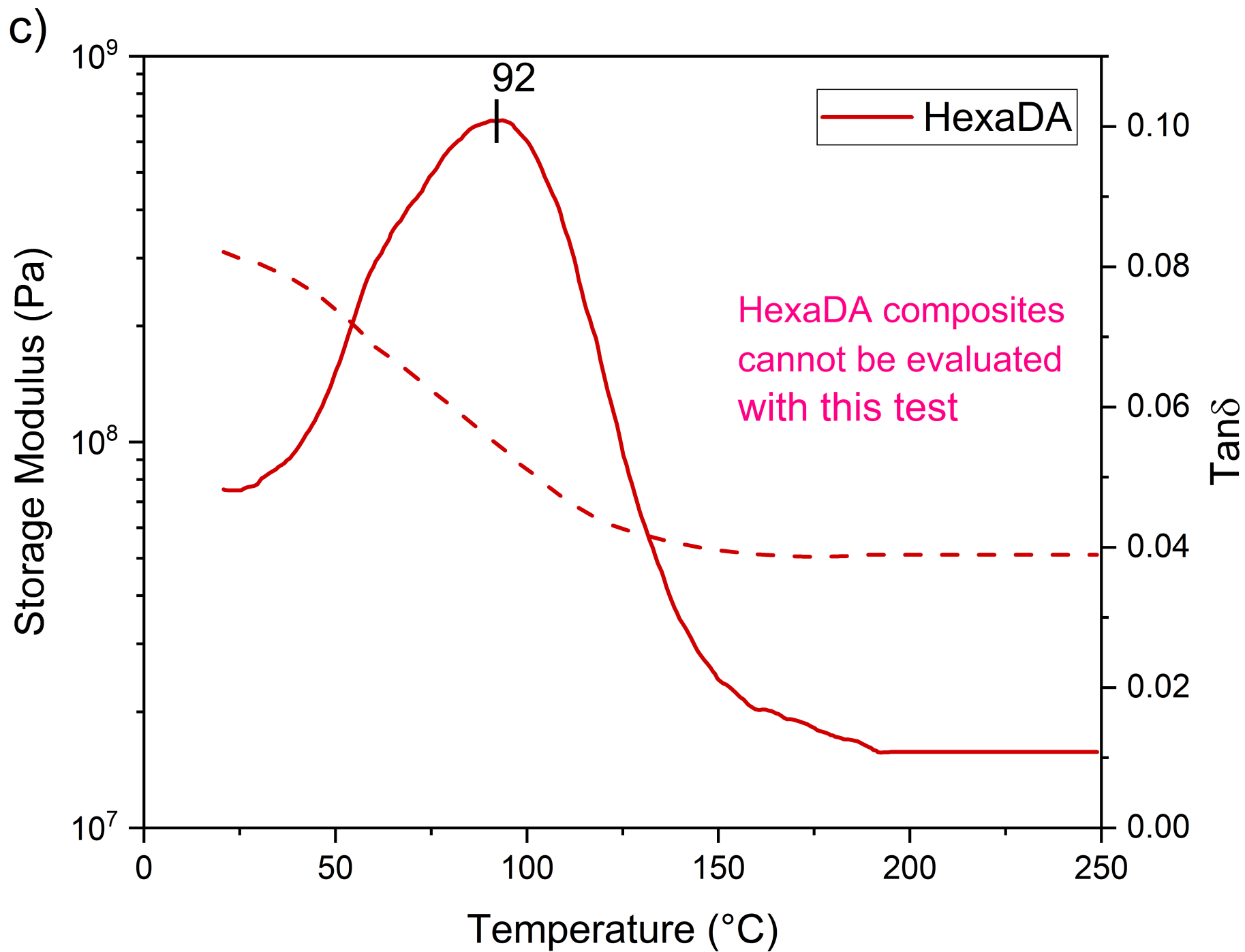

Supplement: Supplementary file 1 [file polymers-15-01633-s001.zip › Fig.S2c.pdf]

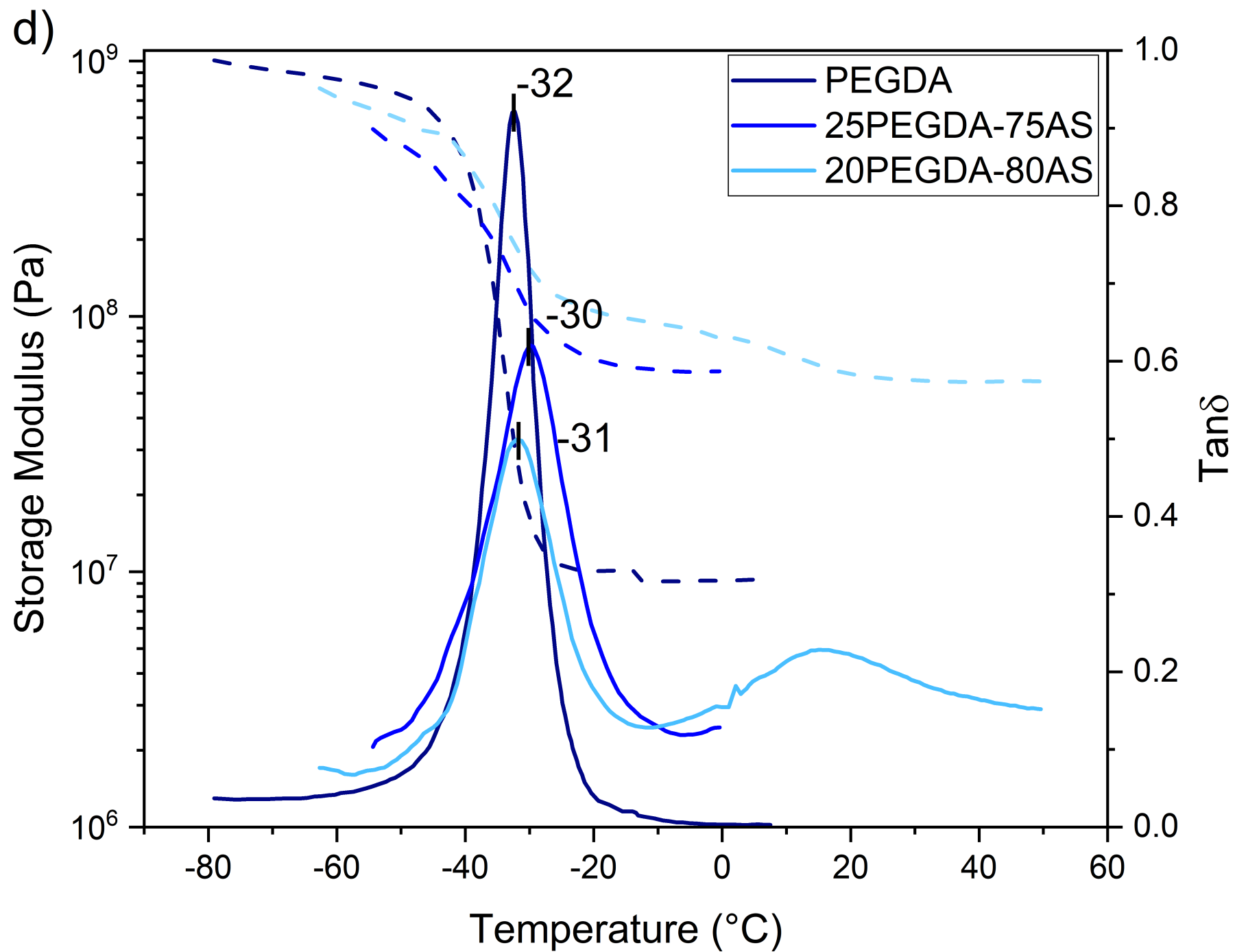

Supplement: Supplementary file 1 [file polymers-15-01633-s001.zip › Fig.S2d.pdf]

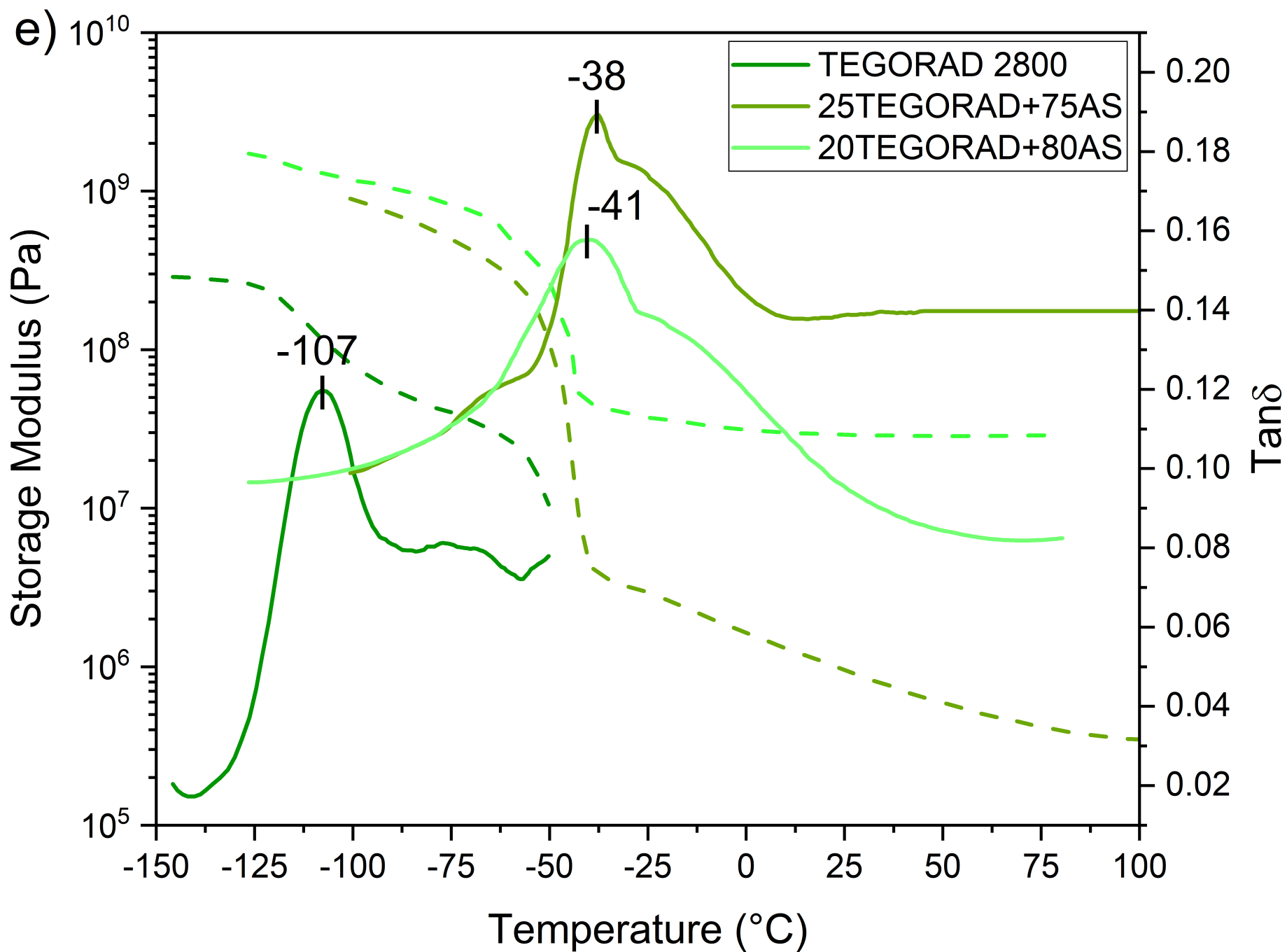

Supplement: Supplementary file 1 [file polymers-15-01633-s001.zip › Fig.S2e.pdf]

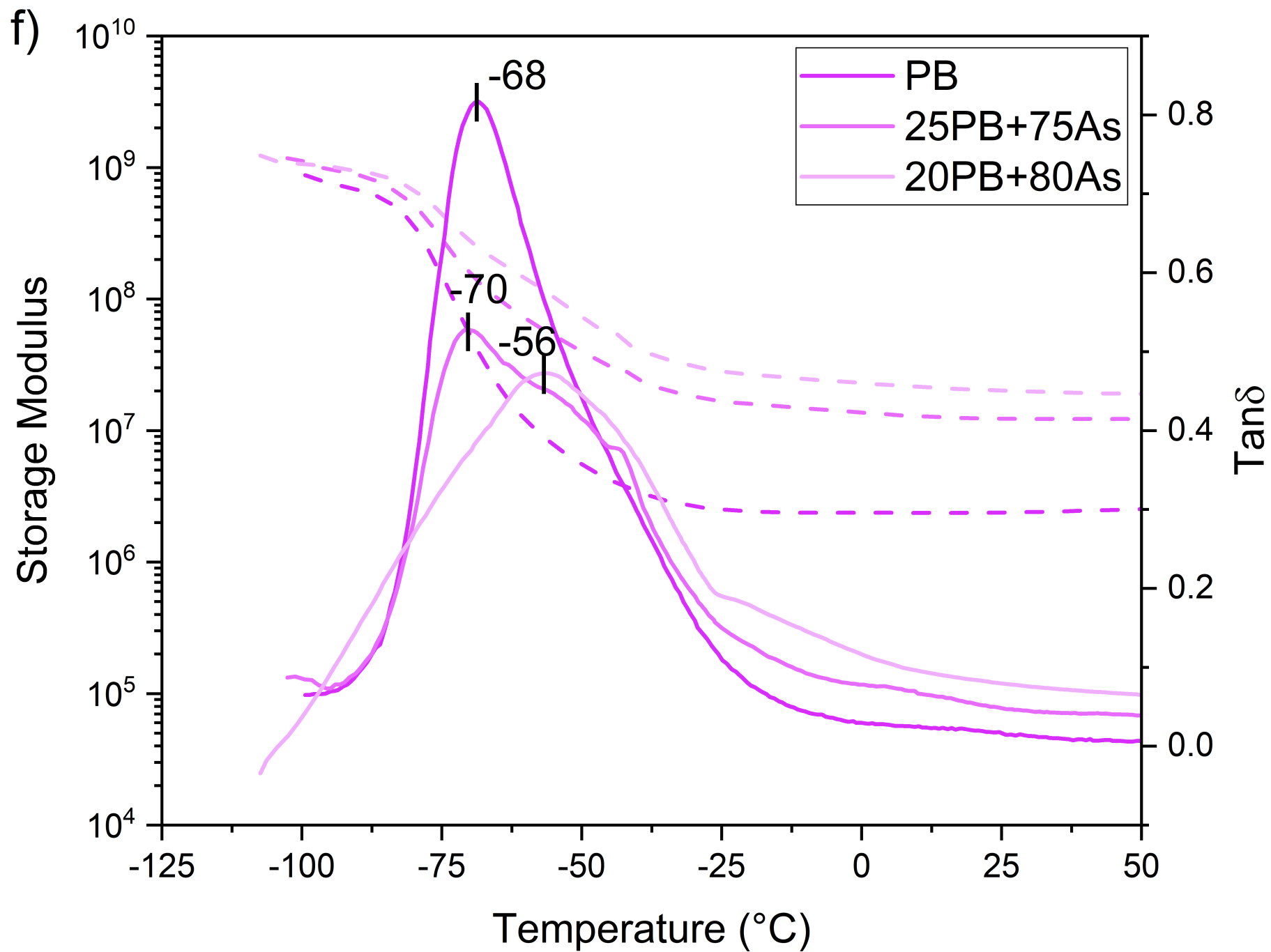

Supplement: Supplementary file 1 [file polymers-15-01633-s001.zip › Fig.S2f.pdf]

a)

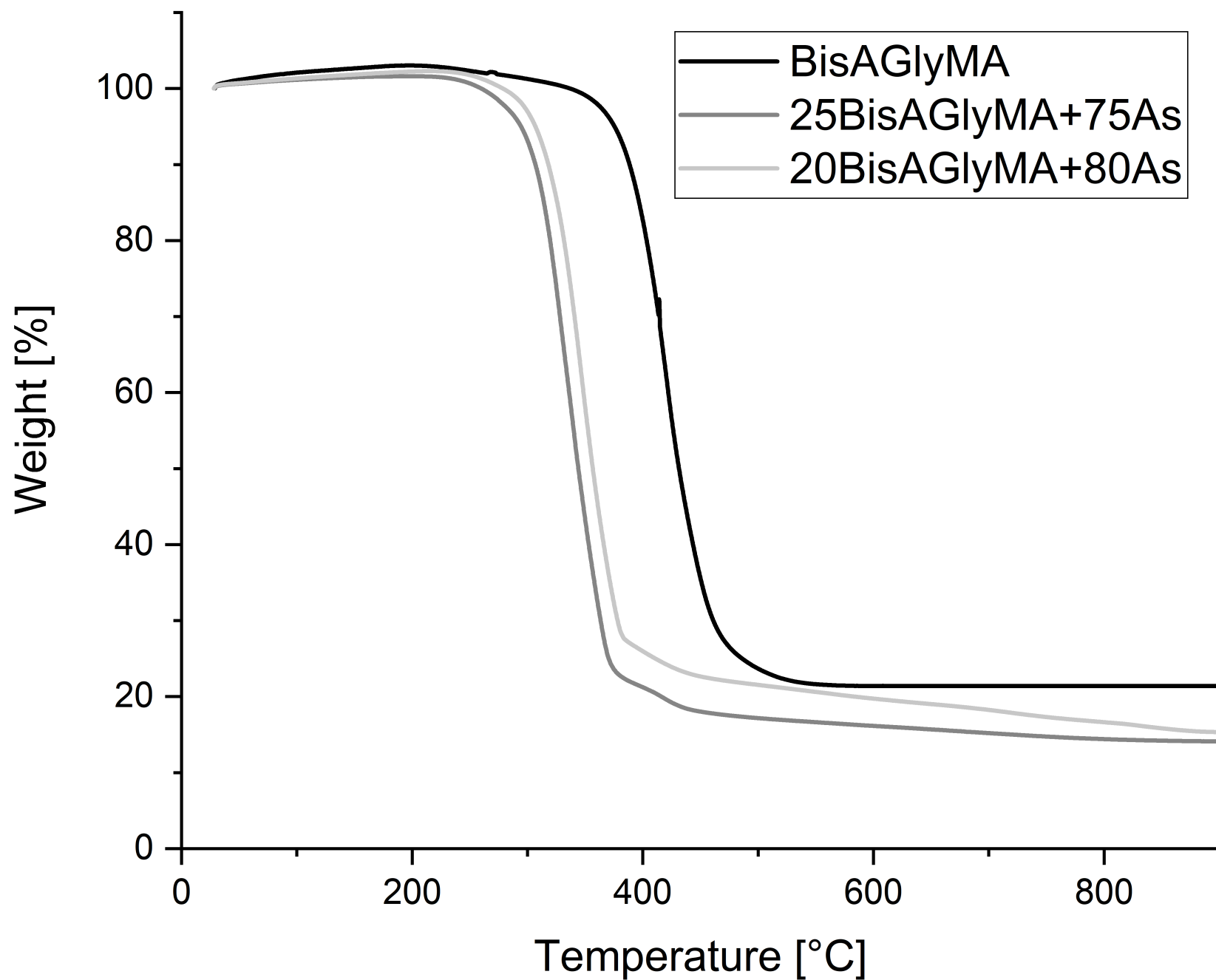

Supplement: Supplementary file 1 [file polymers-15-01633-s001.zip › Fig.S3a.pdf]

b)

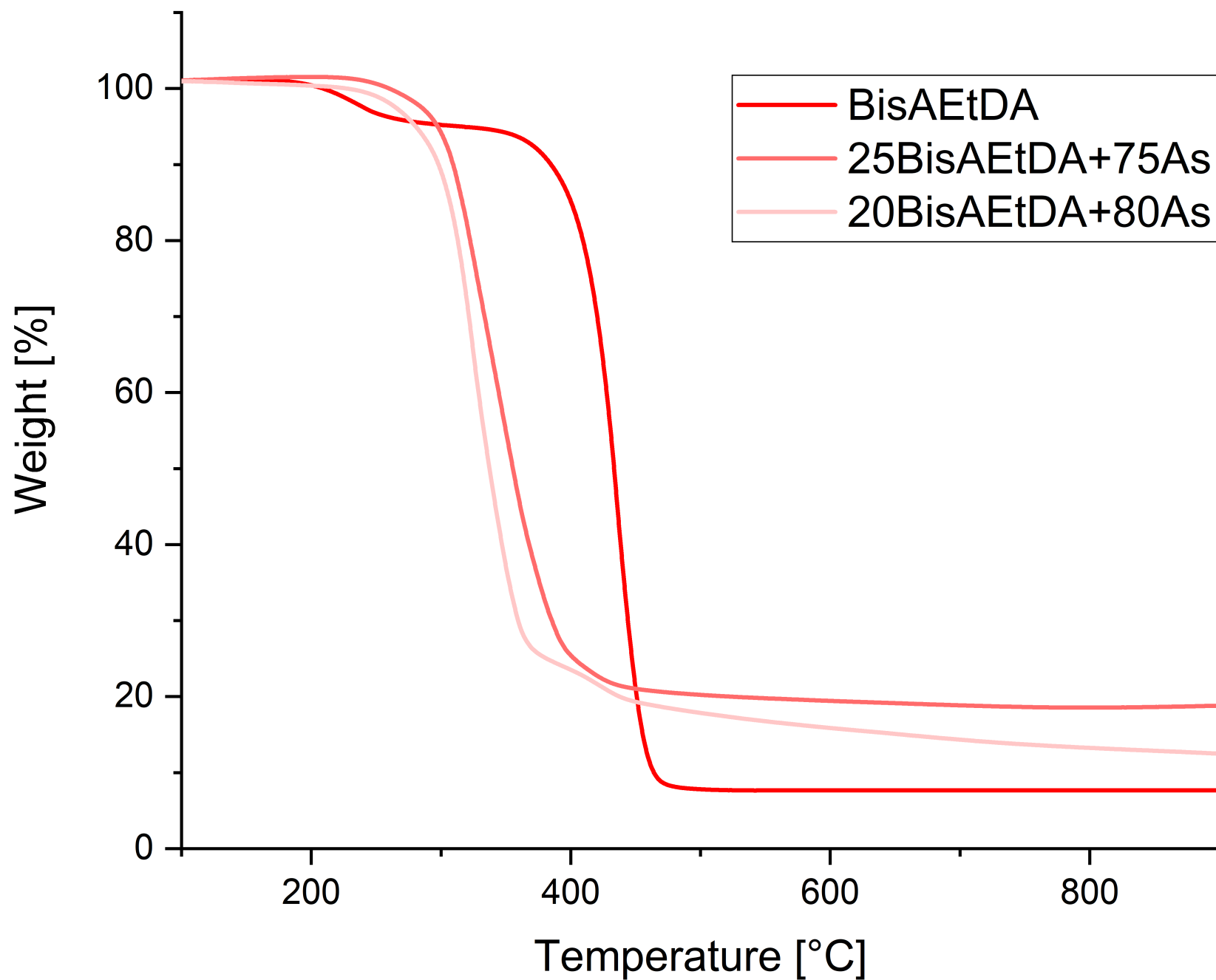

Supplement: Supplementary file 1 [file polymers-15-01633-s001.zip › Fig.S3b.pdf]

c)

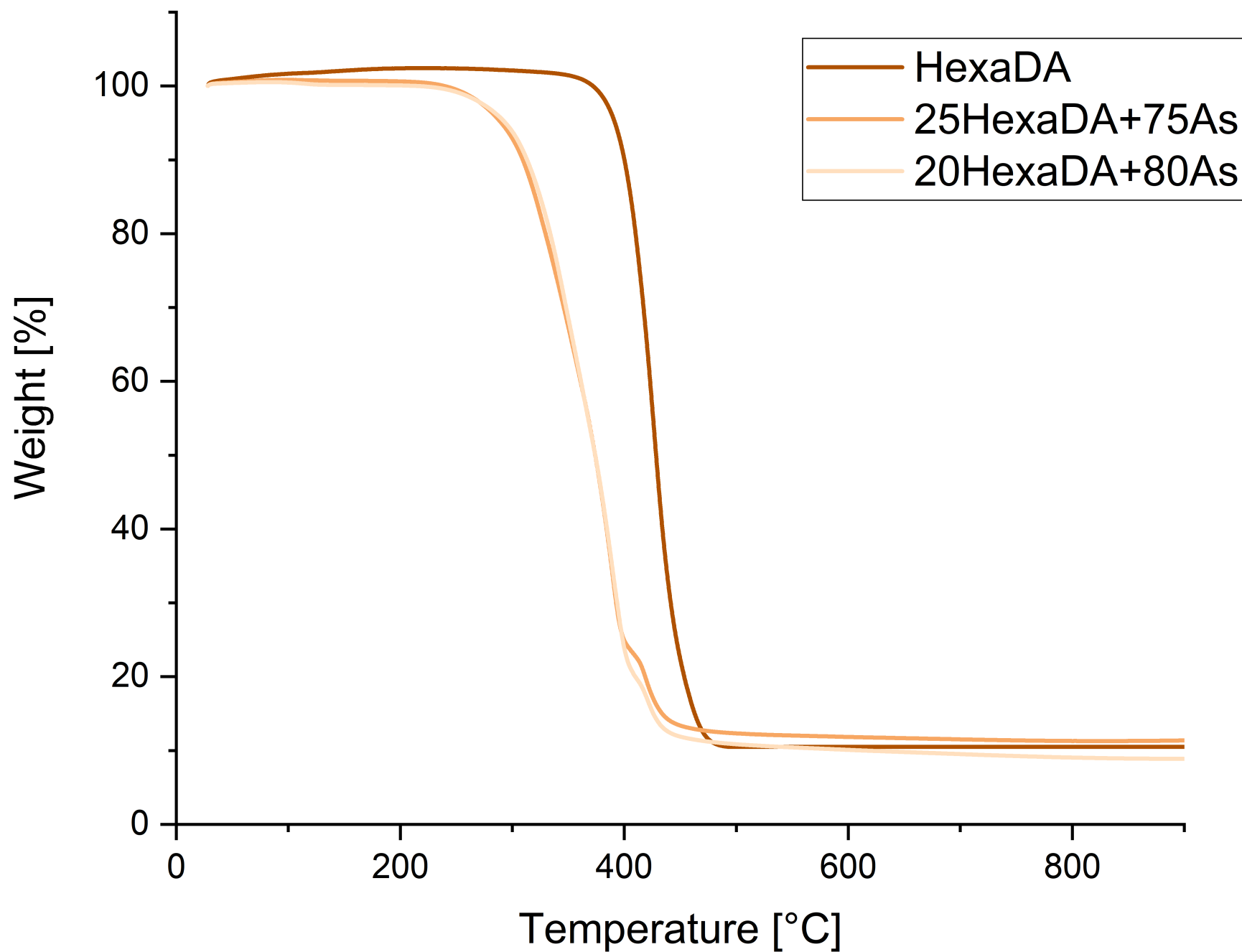

Supplement: Supplementary file 1 [file polymers-15-01633-s001.zip › Fig.S3c.pdf]

d)

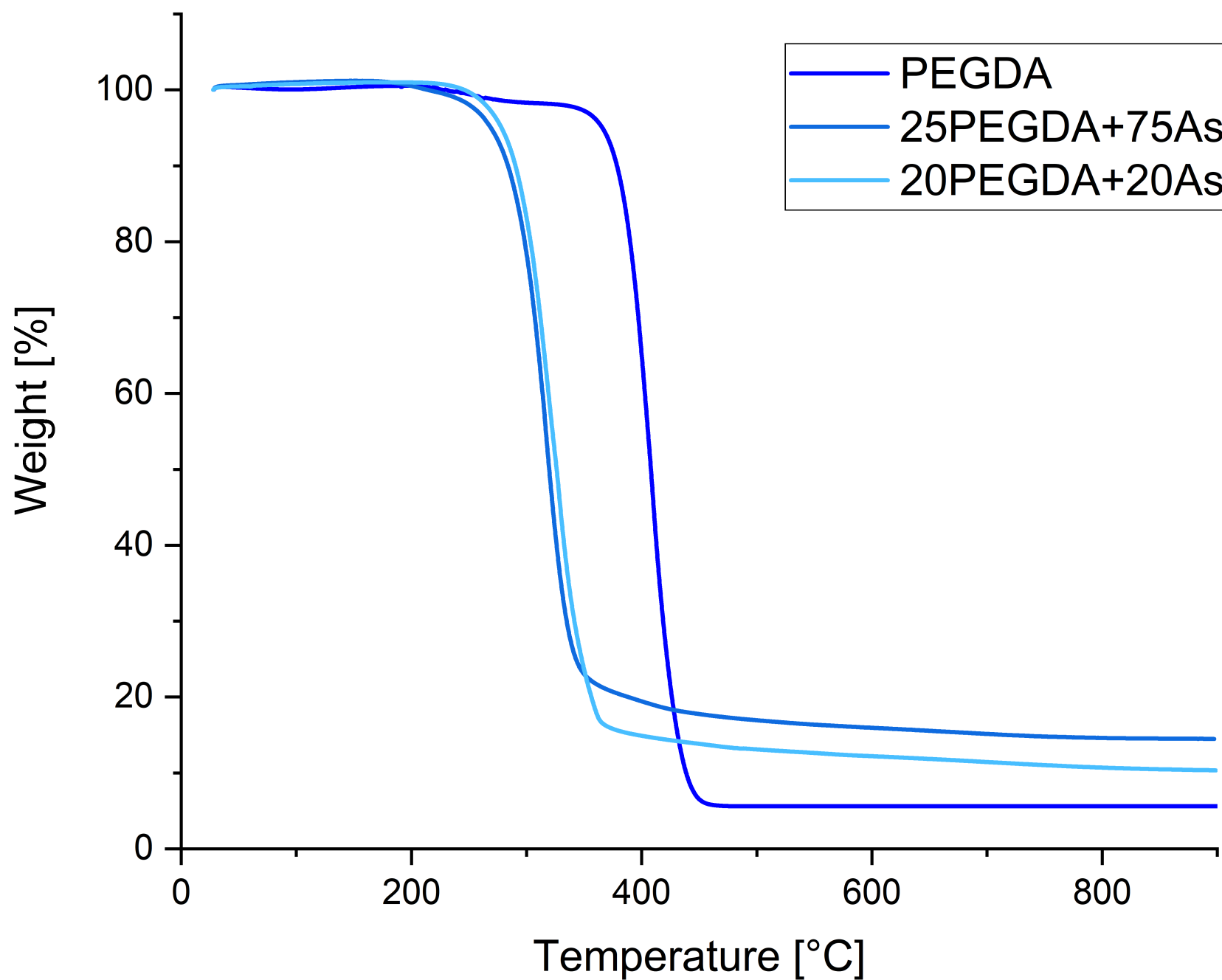

Supplement: Supplementary file 1 [file polymers-15-01633-s001.zip › Fig.S3d.pdf]

e)

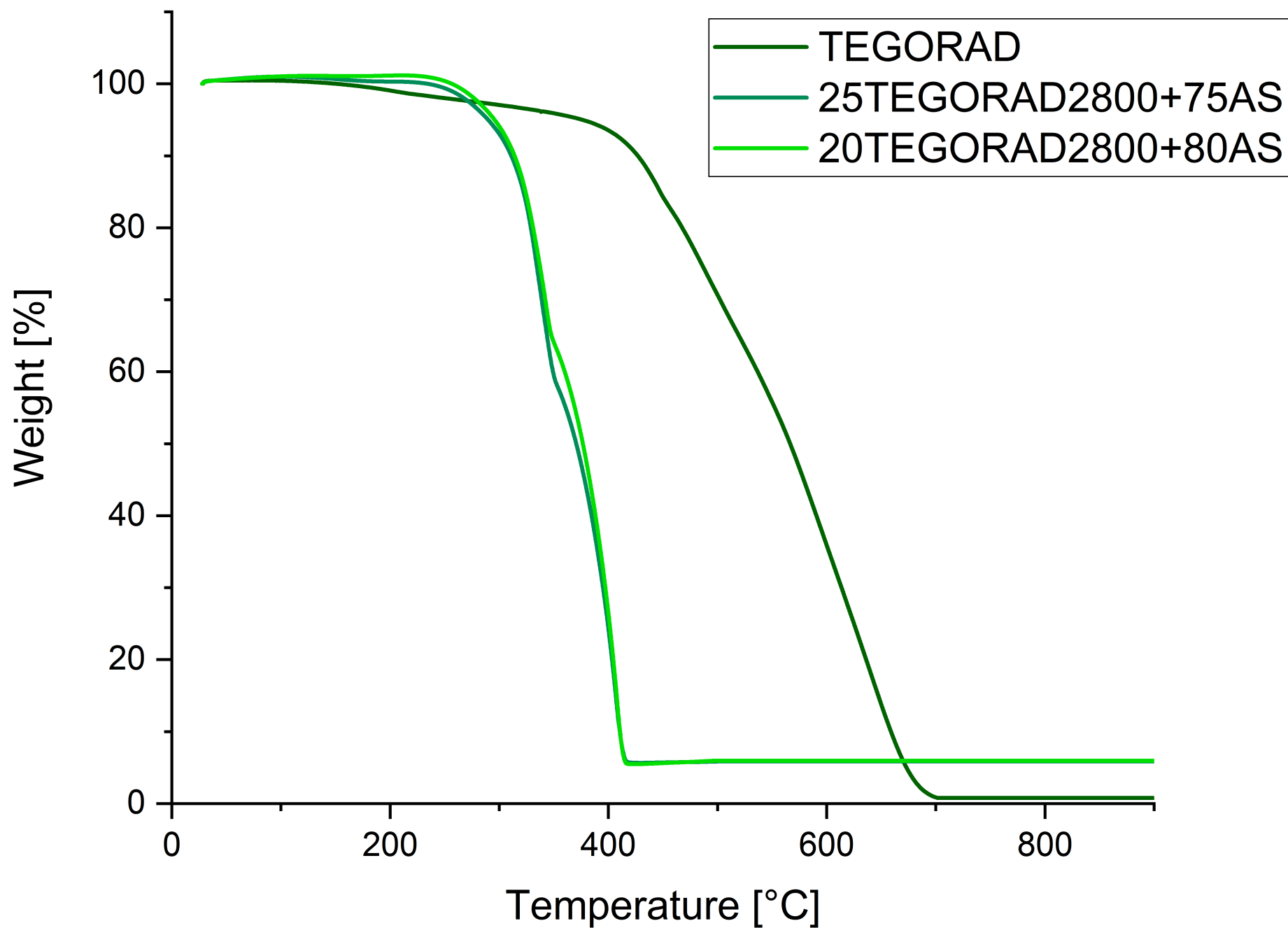

Supplement: Supplementary file 1 [file polymers-15-01633-s001.zip › Fig.S3e.pdf]

f)

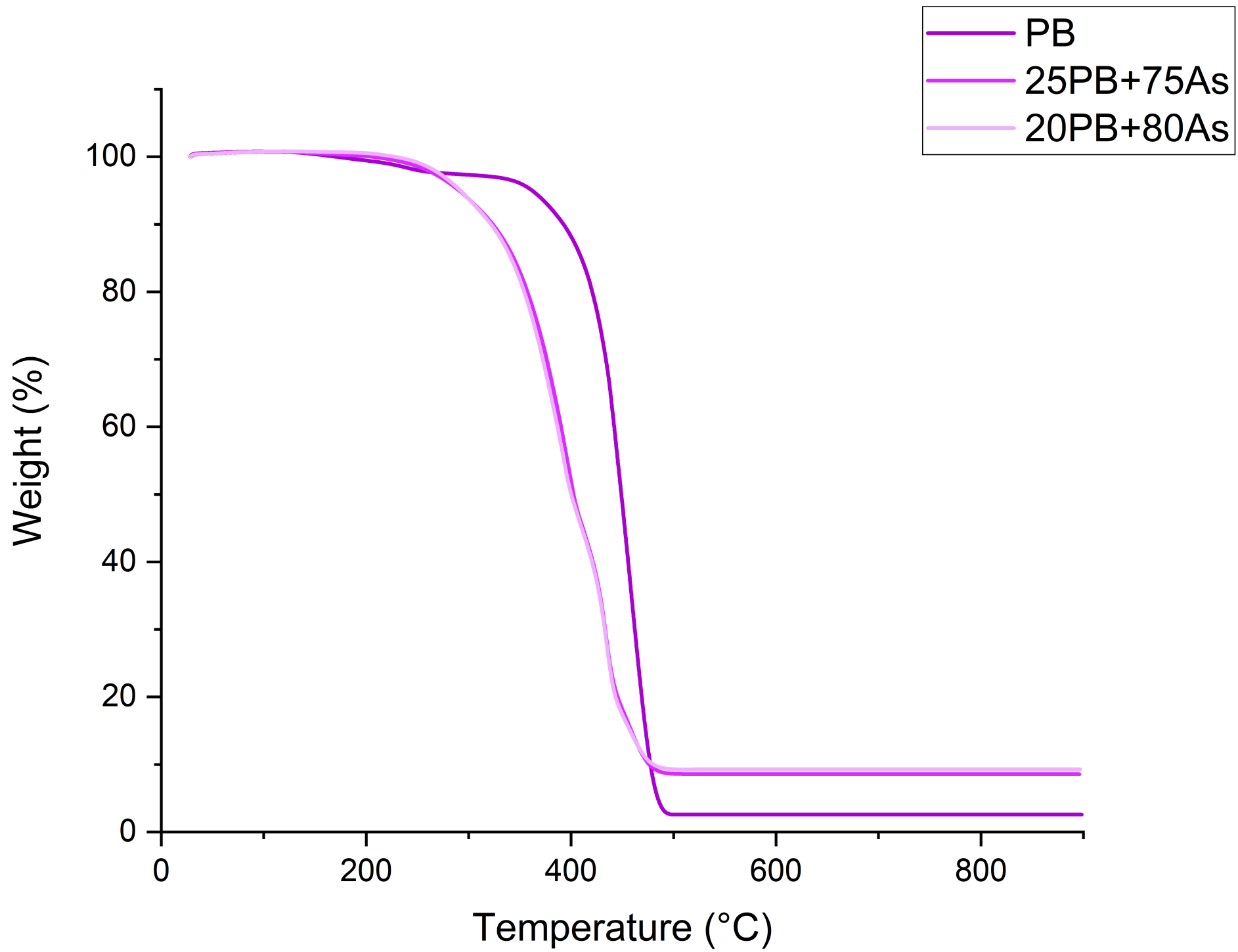

Supplement: Supplementary file 1 [file polymers-15-01633-s001.zip › Fig.S3f.pdf]
